# Proteome Landscapes Decode Organelle Vulnerabilities in cortical and dopaminergic-like induced neurons Across Lysosomal Storage Disorders

**DOI:** 10.1101/2025.10.08.681047

**Published:** 2025-10-08

**Authors:** Felix Kraus, Yuchen He, Yizhi Jiang, Delong Li, Yohannes A. Ambaw, Federico M. Gasparoli, Joao A. Paulo, Tobias C. Walther, Robert V. Farese, Steven P. Gygi, Florian Wilfling, J. Wade Harper

**Affiliations:** 1 Department of Cell Biology, Harvard Medical School, Boston MA, USA; 2 Aligning Science Across Parkinson’s (ASAP) Collaborative Research Network, Chevy Chase, MD 20815, USA; 3 Mechanisms of Cellular Quality Control, Max Planck Institute of Biophysics, Frankfurt, Germany.; 4 Cell Biology Program, Sloan Kettering Institute, New York, NY, USA.; 5 Howard Hughes Medical Institute, New York, NY, USA

**Keywords:** Lysosome, Proteomics, Organelle quality control, LysoIP, iNeuron, iDA, Lysosomal Storage Disorder

## Abstract

Lysosomes maintain cellular homeostasis by degrading proteins delivered via endocytosis and autophagy and recycling building blocks for organelle biogenesis. Lysosomal Storage Disorders (LSDs) comprise a broad group of diseases affecting lysosomal degradation, ion flux, and lipid catabolism. Within this group, sphingolipidoses genes involved in glycosphingolipid breakdown are known (*GBA1*) or candidate (*SMPD1*, *ASAH1*) risk factors for Parkinson’s Disease, though disease mechanisms remain unclear. Using our previously reported LSD mutant proteomic landscape in HeLa cells, we observed pronounced variability in endolysosomal proteome signatures among sphingolipid pathway mutants, with *ASAH1*^−/−^ cells showing altered lysosomal lipid composition, impaired endocytic trafficking, and disrupted ultrastructure by cryo-electron tomography. To extend these findings in a more physiologic context, we generated a human embryonic stem (ES) cell library comprising 23 LSD gene knockouts and profiled proteomic changes during differentiation into cortical and midbrain dopaminergic neurons over a 7 to 10 week period. LSD mutants exhibited lineage-specific alterations in organellar proteomes, revealing diverse vulnerabilities. Notably, *GBA1*^−/−^ and *ASAH1*^−/−^ dopaminergic neurons showed disruptions in synaptic and mitochondrial compartments, correlating with impaired dopaminergic neuronal firing and disrupted presynaptic protein localization. This LSD mutant toolkit and associated proteomic landscape provides a resource for defining molecular signatures of LSD gene loss and highlights convergence of lysosomal dysfunction, synaptic integrity, and mitochondrial health as potential links between sphingolipidoses and PD risk.

Extensive genetic and clinical studies have revealed more than four dozen genes whose inactivating mutation leads to defects in lysosomal function and/or the aberrant accumulation of metabolic intermediates generally referred to as “storage products”^1^. These genes constitute “Lysosomal Storage Disorders” (LSD), a rare (1 in 5,000 live births) set of diseases that affect multiple tissues and organ systems, but are often linked with neurodevelopmental or neurodegenerative phenotypes. Lysosomes are a primary degradative membrane-bound compartment within cells, and contain hydrolases that digest glycoproteins delivered via endocytosis, as well as proteins and organelles delivered via autophagy, thereby facilitating the recycling building blocks^2,3^. Additionally, lysosomes contain enzymes and transporters responsible for recycling of a variety of lipids. As a result of these properties, lysosomes function as important signaling hubs for nutrient availability via the MTOR pathway^2^. LSDs are primarily classified as sphingolipidoses, mucopolysaccharidoses, glycoproteinoses, and neuronal ceroid lipofucinoses (NCL), based on the type of storage material that accumulates, typically within lysosomes (e.g. glycosphingolipids, and cholesterol)^4^. Additional storage material can also accumulate as a secondary response. However, how diverse primary and secondary storage material accumulation interfaces with distinct multisystem disease phenotypes is poorly understood.

Sphingolipidoses occurs upon defective breakdown of sialic-acid-containing glycosphingolipids (Extended Data Fig. 1a), which are highly abundant in the nervous system. Gangliosides are via endocytosis trafficked from the plasma membrane to intralumenal vesicles (ILVs) formed within endolysosomes. Sphingolipid catabolism is thought to occur, in part, on ILVs. Current models posit that negatively charged bis(monoacylglycerol)phosphate (BMP) lipids, present in the outer leaflet of ILVs, function to recruit positively-charged enzymes that participate in ganglioside breakdown to the limiting ILV membrane^5–8^. Defects in ganglioside breakdown cause neurological diseases, for example, upon loss of the hexosaminidase *HEXA* that converts GM2 to GM3 (Tay-Sachs disease), or upon loss of *GRN*, where decreased BMP levels result in increased GM2 and other sialyated gangliosides (Frontotemporal dementia, FTD)^7^. The penultimate product in ganglioside breakdown – ceramide – is generated from a glucosylceramide intermediate by a lysosomal glucocerebrosidase GBA1 and independently from sphingomyelin by lysosomal SMPD1 (sphingomyelin phosphodiesterase)^9^ (Extended Data Fig. 1a). Homozygous loss-of-function *GBA1* mutations are causal for Gaucher’s disease, while heterozygous mutations increase the risk for Parkinson’s disease (PD)^10–14^. Similarly, *SMPD1* variants cause Niemann-Pick disease type A/B and have also been implicated in PD^15–18^. Breakdown of ceramide to the terminal products sphingosine and fatty acid is performed by ASAH1, a lysosomal acid ceramidase whose mutation underlies Farber’s disease^19^. As with *GBA1* and *SMPD1*, *ASAH1* has been proposed to be a risk factor for PD^20^. Thus, the propensity of defects in glycosphingolipid in disease, including potential PD risk factors, pinpoints this pathway as a central component of endolysosomal and cellular homeostasis.

One approach to understanding the complexity of LSDs involves comparative molecular profiling of cells lacking a cross-section of LSD genes in diverse functional categories, as we recently reported in the context of HeLa cells^21^. Here, we extend this approach in two distinct ways. First, proteome correlation analysis of endolysosomal and autophagy signatures in HeLa cells lacking LSD proteins revealed an unexpectedly broad variability in those cells lacking enzymes in the sphingolipid degradation pathway, with *ASAH1*^−/−^ cells displaying the largest variation. Proteomic analysis of lysosomes from *ASAH1*^−/−^ HeLa cells revealed increased abundance of early and recycling endosomal proteins, as well as ER proteins, and this was accompanied by a reduction in trafficking of the endocytic cargo transferrin (Tf). Moreover, we observed alterations in the ultrastructure of endolysosomes as examined by cryo-electron tomography (cryo-ET), which correlated with altered abundance of sphingolipid and BMP lipids in lysosomes. In a second approach, we created a library of human embryonic stem (ES) cell lines lacking one of 23 LSD genes from sphingolipidoses, NCL, and integral membrane protein disorder classes, and performed longitudinal proteomics during differentiation to either cortical-like induced Neurons (iNs) or midbrain dopaminergic neurons (iDAs). We demonstrate the power of this LSD mutant toolkit through detailed analysis of proteomes of selected genotypes at day 50 of differentiation and up to day 70 of differentiation in Control and *ASAH1*^−/−^ cells. This led to the identification of distinct patterns of protein abundance correlations associated with specific organelles in iN and iDA cells, including dramatic effects on synaptic and mitochondrial compartments observed in *GBA1*^−/−^ and *ASAH1*^−/−^ cells. Ultrastructural analysis of neuronal projections indicated defects in *ASAH1*^−/−^ iDA cells that correlated with reduced neuronal firing of iDA, but not iN, cells under basal conditions and with altered localization of pre-synaptic proteins bassoon (BSN) and synaptophysin (SYP). In summary, this study underscores the utility of this stem-cell line toolkit and proteomic data in elucidating molecular signatures associated with LSD loss of function mutants across cells and organelles. Additionally, our data suggest that vulnerabilities in mitochondrial or synaptic functions – both of which are associated with PD^20,22,23^ – are linked with mutations in *GBA1* and *ASAH1*.

## RESULTS

### Proteomic and lipidomic evaluation of the early endosomal system in *ASAH1*^−/−^ HeLa cells

**Extended Data Fig. 1:**
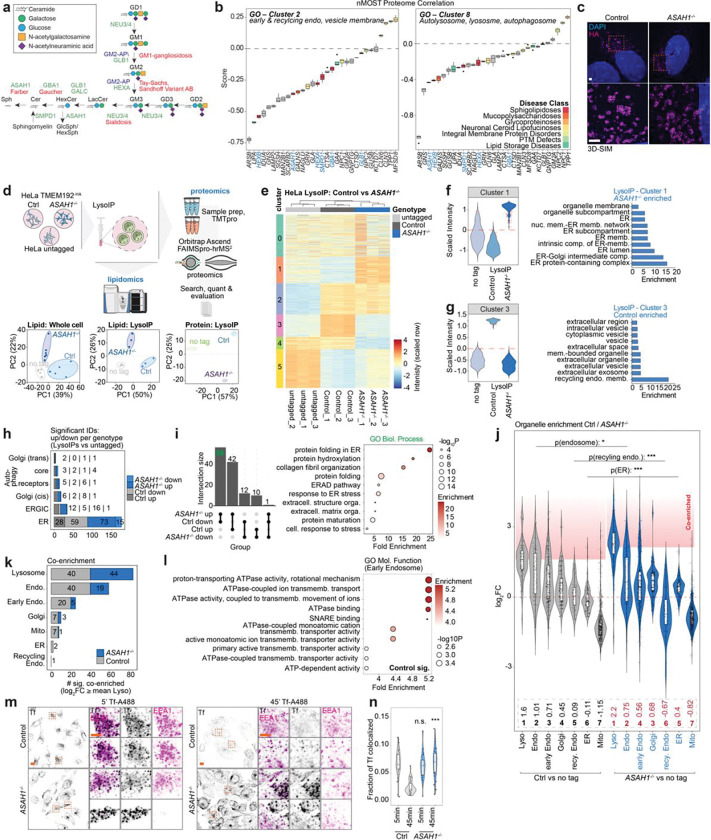
Proteomic and lipidomic analysis of HeLa cells with or without the acid ceramide hydrolase ASAH1. **a**, Scheme depicting the pathway for Sphingolipid catabolism, including steps involving ASAH1, which converts ceramide to sphingosine and fatty acids. Modified from our previous publication^7^. **b**, Ranked proteome correlations based on nMOST HeLa data^21^ for early and recycling endosomes (left) and lysosomes and autophagy (right). Blue labels indicate genotypes belonging to the Sphingolipidoses class of LSDs. **c**, Example 3D-SIM images of Control and *ASAH1*^−/−^ HeLa cells immunostained with for α-HA (TMEM192^HA^). DAPI (blue) was used to mark nuclei. Scale bar: 2 μm. **d**, Schematic for TMTpro proteomics and label-free lipidomic of LysoIP samples from *ASAH1*^−/−^, Control and untagged HeLa cells. PCA plots are shown below. **e**, Heatmap of TMTpro-based proteomics of untagged, Control and *ASAH1*^−/−^ LysoIP samples from HeLa cells. Sample clusters are labeled on the left. Relative TMT intensities (log_2_FC) are indicated in the red/blue color scale. **f**, Violin plot and corresponding GO enrichment of proteins IDs belonging to cluster 1 (*ASAH1*^−/−^ LysoIP enriched). **g**, Violin plot and corresponding GO enrichment of proteins IDs belonging to cluster 3 (Control LysoIP enriched). **h**, Bar plot of significant enriched (up) and de-enriched (down) protein IDs in Control and *ASAH1*^−/−^ LysoIPs per organelle annotations. **i**, Left: upset plot of data from panel **h**, showing overlap between annotations. Right: GO enrichment analysis for biological processes from intersection-pair (*ASAH1*^−/−^ up and Ctrl down). **j**, Violin plots depicting log_2_FC of selected organelles and sorted by abundance. Average enrichment and rank based on Control LysoIP is plotted under each violin. Red-shaded area is considered “co-enriched” for analysis purposes. LysoIP(Control vs *ASAH1*^−/−^): endosome: *P(0.04); recycling endosome: ***P < 0.0001; ER: ***P < 0.0001.**k**, Bar plot of number of “co-enriched” IDs per annotation. **l**, GO enrichment analysis for molecular function of early endosome IDs from panel **k**. **m**, Images of HeLa *ASAH1*^−/−^ and Control cells incubated for the indicated times with Alexa-488 conjugated Transferrin (Tf-A488, gray scale) and immunofluorescently labeled for EEA1 (α-EEA1, magenta). Scale bar: 10 μm and 5 μm. **n**, Quantification of panel **m**. based on three replicates (per genotype and staining) containing 9 randomly selected ROIs each.

**Fig. 1:**
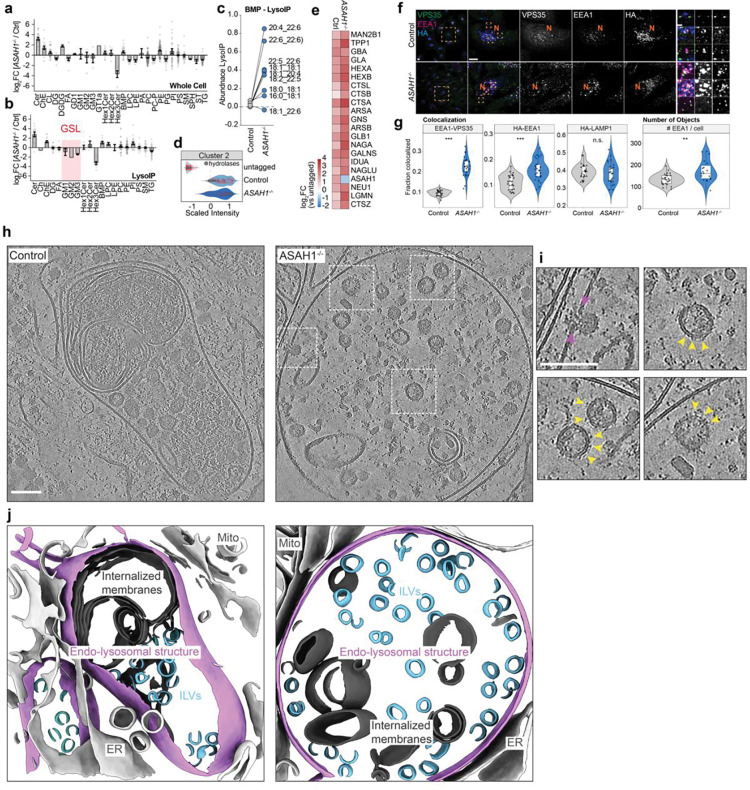
Loss of *ASAH1*^−/−^ disturbs lysosomal lipidome and endo-lysosomal morphology. **a**, Log_2_FC of *ASAH1*^−/−^ vs Control whole cell lipidomics from HeLa cells. Data based on n=5 biological replicates per genotype. **b**, Log_2_FC of *ASAH1*^−/−^ vs Control LysoIP lipidomics from HeLa cells. Data based on n=3 biological replicates per genotype. **c**, Lineplot of select BMP species in Control and *ASAH1*^−/−^ LysoIP samples. Data based on n=3 biological replicates per genotype. **d**, Violin plot of cluster 2 of heatmap (Extended Data Fig. 1e) depicting enrichment of proteins of Control and *ASAH1*^−/−^ over untagged cells. Lysosomal hydrolases are shown as grey circles. **e**, Log_2_FC of selected lysosomal hydrolases based on LysoIP proteomics of Control or *ASAH1*^−/−^ cells vs untagged cells. TMTpro proteomics, based on n=3 biological replicates per genotype. **f**, Confocal images of HeLa *ASAH1*^−/−^ and Control cells immunofluorescently labeled for VPS35, EEA1 and HA (TMEM192). N; nuclear space. Scale bar: 10 μm and 2 μm (zoom ins). **g**, Evaluation of panel **f** measuring colocalization and number of indicated objects. EEA1-VPS35: ***P< 0.0001; HA-EEA1: ***P < 0.0001; # EEA1 / cell: **P(0.008). **h**, Example tomogram of Control and *ASAH1*^−/−^ endo-lysosomal structures. Scale bar 100 nm. **i**, Zoom ins of white boxes in panel **h**, showing inward budding membrane events (purple arrowheads) and ILVs studded with glycosylated proteins (yellow arrowheads). Scale bar 100 nm. **j**, 3D-renderings of tomogram segmentations from panel **h**.

Given the relationship between lysosomal storage disorders and the endolysosomal system, we mined our previous HeLa cell proteomic data across more than two dozen LSD mutations^21^ for signatures representing alterations in early/recycling endosomes and lysosomes (Gene Ontology Clusters 2 and 8) (Extended Data Fig. 1b). Unexpectedly, components of the sphingolipid catabolism pathway displayed differential often negative correlations with disturbance in the endosomal and lysosomal proteome, with *ASAH1*^−/−^ and *HEXA*^−/−^ cellular proteomes displaying the greatest anti-correlation. ASAH1 has historically been viewed as a lysosomal-localized enzyme, as annotated in Uniprot. However, we previously detected ASAH1 in early endosomes in cancer cell lines, as isolated by Endo-IP using EEA1 (early endosomal antigen 1) as the affinity handle ^24^, suggesting its localization in less mature endolysosomal compartments. To our knowledge, the proteome and lipidome of lysosomes from *ASAH1*^−/−^ cells have not been molecular profiled in any system. This together with the potential link between ASAH1 and PD led us to performed LysoIP from Control and *ASAH1*^−/−^ HeLa cells containing an endogenously tagged TMEM192^HA^ locus^21^ along with parental HeLa cells as a negative control. Isolated lysosomes and whole cell extracts were subjected to lipidomics and TMT-based proteomics (Extended Data Fig. 1c,d; **Supplementary Table 1,2**). Ceramide species (Cer) increased ~3-fold in abundance in both lysates and purified lysosomes from *ASAH1*^−/−^ cells, consistent with *ASAH1* deficiency, and this was mirrored by a ~4-fold reduction in Hex3Cer species (Fig. 1a,b; **Supplementary Table 1**). Interestingly, several BMP species were increased in abundance in lysosomes in the absence of *ASAH1* (Fig. 1c), which may underlie the modest (~2-fold or less) reduction in glycosphingolipids (GSL) GM1, GM2, GM3 in lysosomes^7^ (Fig. 1a,b).

Proteomic analysis of LysoIP samples from Control and *ASAH1*^−/−^ cells revealed alterations in composition of quantified and annotated endosomal and lysosomal proteins, with 6 clusters identified (Extended Data Fig. 1e; **Supplementary Table 2**). As expected, numerous endolysosomal proteins (Cluster 2) were increased in abundance in Control and *ASAH1*^−/−^ cells relative to parental cells lacking the LysoIP handle, and this included several lumenal lysosomal enzymes that were also modestly increased in abundance in *ASAH1*^−/−^ lysosomes (Fig. 1d,e). In contrast, we identified additional clusters with strongly differential protein levels: Cluster 1 reflected proteins enriched in lysosomal fractions from *ASAH1*^−/−^ cells, which included GO terms related to endoplasmic reticulum (ER), while Cluster 3 reflected a reduced level of proteins linked with recycling endocytic membrane GO terms (Extended Data Fig. 1f,g). We then determined the number of proteins significantly altered in *ASAH1*^−/−^ LysoIP samples across organelle categories. We found that ER proteins represent the annotation class with the largest number of proteins identified as changing in abundance (up or down) in LysoIP samples from *ASAH1*^−/−^ cells, as compared with Control cells (Extended Data Fig. 1h,i). We then examined the number of “co-enriched” proteins (proteins ≥ mean lysosomal enrichment) across compartments. Besides enrichment of lysosomal and endosomal proteins, loss of *ASAH1* resulted in aterations in “co-enrichment” profiles of other compartments (Extended Data Fig. 1j). Violin plots of relative co-enrichment across multiple organelles revealed an overall increase in the mean log_2_FC for ER proteins in *ASAH1*^−/−^ cells relative to that seen in Control cells and a corresponding reduction in mean log_2_FC for proteins linked with recycling endosomes, consistent with cluster analysis. Numerically speaking, while no differences were observed in lysosomal annotation, endosomal and early endosomal compartments were de-enriched when compared with lysosomal annotations in *ASAH1*^−/−^ cells (Extended Data Fig. 1k). The early endosome category was enriched in molecular function GO-term proteins linked with proton transport via the vATPase (Extended Data Fig. 1l). Consistent with defects in endolysosomal trafficking and/or maturation, we found that *ASAH1*^−/−^ HeLa cells displayed an increased co-localization of the endosomal recycling Retromer subunit VPS35 and the early endosome marker EEA1, as well as an increase in the co-incidence of EEA1 and the TMEM192^HA^ lysosomal tag when compared with Control cells (Fig. 1f,g). The number of EEA1-positive puncta was modestly increased (p<0.05), while the overlap of TMEM192^HA^ and LAMP1 was not statistically different (Fig. 1f,g). Moreover, Transferrin (Tf) was readily cleared from EEA1-positive structures in Control cells but remained elevated in *ASAH1*^−/−^ cells 45 min post Tf treatment (p<0.001) (Extended Data Fig. 1m,n). Taken together, these data indicate that loss of *ASAH1* in HeLa cells promotes defects in endolysosomal maturation/recycling, and a propensity of lysosomes to remain associated with ER proteins during LysoIP.

### Visualization of *ASAH1*^−/−^ endolysosomal compartments by cryo-ET

**Extended Data Fig. 2:**
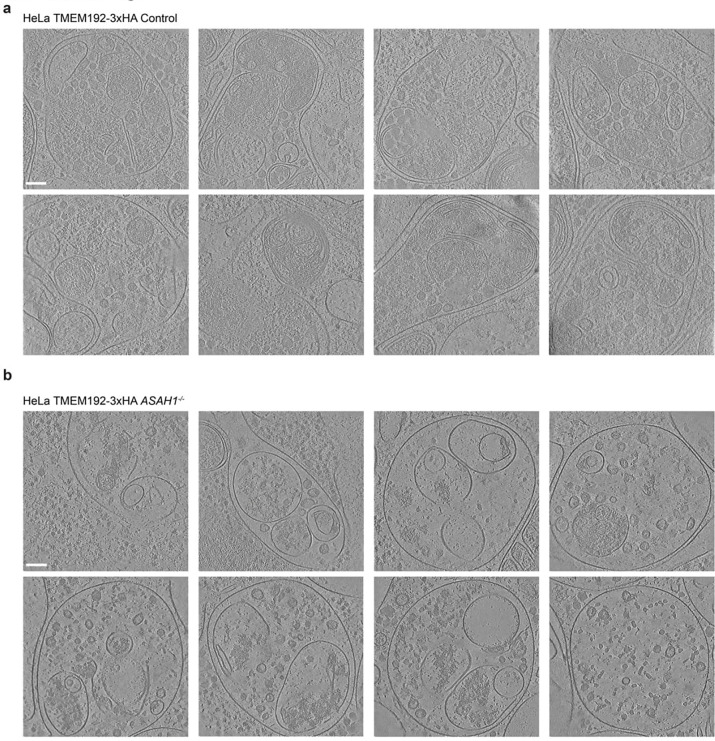
Analysis of Control and *ASAH1*^−/−^ HeLa cells by cryo-ET. **a**, Gallery of representative tomograms of HeLa Control lysosomes. Scale bar 100 nm. **b**, Gallery of representative tomograms of Hela *ASAH1*^−/−^ lysosomes. Scale bar 100 nm.

The alterations in the endolysosomal system in *ASAH1*^−/−^ HeLa cells led us to examine organelle ultrastructure *in situ* by cryo-focused ion beam (cryo-FIB) milling paired with cryo-ET (Fig. 1h,i). Endolysosomal structures in Control cells have typical ultrastructural features of auto-/endolysosomes, including frequent single or double membrane structures within the limiting membrane and dense material indicative of degradative cargo (Fig. 1h, Extended Data 2a,b). Endolysosomal structures in *ASAH1*^−/−^ cells appeared swollen compared to those in Control cells, which showed dense packing of cellular material (Fig. 1h). Notably, membranous material was largely absent in endolysosomal structures in *ASAH*^−/−^ cells. In one tomogram of *ASAH1*^−/−^ cells, we observed an inward-budding vesicle within an endolysosome (Fig. 1h,i), along with numerous intraluminal vesicles (ILVs) that appeared to be studded with glycosylated proteins (Fig 1h, i). Segmentation of tomograms highlights the difference in size and membrane structure packing within the endolysosomes of Control or *ASAH1*^−/−^ cells, and reveals the close proximity of ER to the limiting membrane (Fig. 1j and **Supplementary Movies 1** and 2). Although difficult to quantify due to the swollen nature of endolysosomes in *ASAH1*^−/−^ cells, the appearance suggests increased ER contacts with the endolysosomal limiting membrane, potentially explaining the increase in ER proteins identified in *ASAH1*^−/−^ LysoIP proteomics compared with Control cells (Fig.1j and Extended Data Fig. 2f). Taken together our proteomic and imaging analysis is consistent with dysregulation of endolysosomes in *ASAH1*^−/−^ cells with an increase in early endosomal character of TMEM192^HA^-containing lysosomes (LysoIP), possibly reflecting numerous ILVs present in *ASAH1*^−/−^ endolysosomes (Fig. 1j).

### A stem cell tool kit for systematic analysis of LSD genes

**Extended Data Fig. 3:**
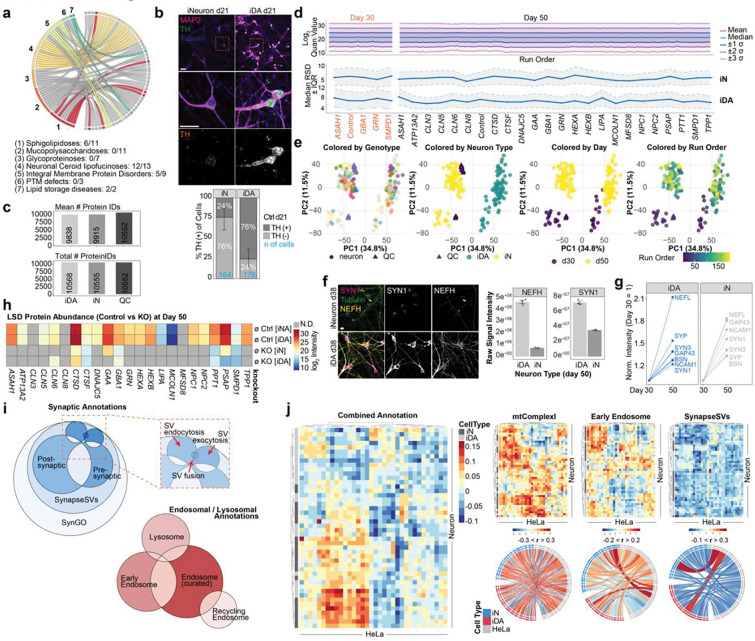
Proteome atlas of lysosomal Storage Disorder neuronal cell models. **a**, Circos plot of LSD deletion coverage in human ES cells. LSD gene editing campaign focused on three disease-classes (Sphingolipidoses, NCL and Integral Membrane Protein Disorders). **b**, Top: Confocal images of Control iN and iDA neurons at day 21 of *in vitro* differentiation, labeled for MAP2 (magenta), TH (green) and Tubulin (blue). Scale bar: 10 μm. Bottom: evaluation of % TH (+) neurons in iN or iDA (n(cells): iN = 164; iDA = 176). **c**, Average and total number of quantified IDs per sample across neuron types for day 50. **d**, Top: Lineplot of mean, median and sigma (range: 1–3) of log_2_ quant value across run order for day 30 and day 50 cohort. Bottom: As above, but plotted for median RSD ± IQR for iN (top) and iDA (bottom). **e**, PCA plots of total proteome datasets, color by genotype, neuron type, day of differentiation and run order. **f**, Left: Confocal images of Control iN and iDA neurons at day 38 of differentiation, immunostained for SYN1 (magenta), NEFH (yellow) and Tubulin (green). Scale bar: 10 μm. Right: Proteomic evaluation of SYN1 and NEFH abundance in iDA cells at day 50 of differentiation. **g**, Line plot depicting intensity of select neurogenesis markers on day 30 and day 50 in iN and iDA neurons from Control cells (normalized to day 30). **h**, Heatmap of target LSD protein abundance in Control and deletion mutants for iN and iDA. See Supplementary Table 1. **i,** Eulerplot depicting the overlap and scope of synaptic and endosomal/lysosomal annotations used for this study. Proteins in each category are listed in Supplementary Table 1. **j**, Left, Correlation heatmap of nMOST Hela dataset^21^ (x-axis) vs. nDIA neuron (y-axis). Right, Three examples for annotations with high, medium and low correlation are plotted on the right.

The analysis of *ASAH1*-deficient cells, together with our previously analysis of *NPC1* and *NPC2*-deficient cells^21^, revealed diverse alterations in the ultrastructure of endolysosomes, as well as cellular lipidome and proteome components in HeLa cells. To systematically examine potential alterations in the properties of organellar systems more broadly and in a more physiologically relevant context, we sought to develop a stem cell-based LSD toolkit across diverse LSD genes. We employed an H9 human embryonic stem cell (hESC)-based system^21,25^ containing an inducible AAVS1-NGN2 cassette for efficient neuronal differentiation and endogenously tagged TMEM192–3xHA (heterozygous) for LysoIP (H9^NGN2;TMEM192-HA^). CRISPR-Cas9 ribonuclear particles were programmed with gRNAs designed to create inactivating indels in 23 LSD genes implicated in sphingolipidoses (*GBA1*, *ASAH1*, *HEXA*, *HEXB*, *PSAP*, *SMPD1*), NCL (*CLN1*, *CLN2*, *CLN3*, *DNAJC5*, *CLN5*, *CLN6*, *CLN7*, *CLN8*, *ATP13A2*, *GRN*, *CTSD*, *CTSF*), LSD gene products that reside in the lysosomal membrane (*MCOLN1* (encoding TRMPL1), *NPC1*, *NPC2*), as well as *LIPA* (Wolman’s disease) and *GAA* (Pompe’s disease) and then transfected into parental H9^NGN2;TMEM192-HA^ cells (referred to hereafter as Control cells) (Extended Data Fig. 3a; Supplementary Table 3). Homozygous clones with frameshift indels were identified by locus sequencing (Supplementary Table 3). We also confirmed the ability of parental Control cells to efficiently convert to cortical-like iNeurons (iN) or midbrain-like dopaminergic neurons (iDA) using established methods^26,27^ by immunofluorescence with α-MAP2 and α-TH (Tyrosine Hydroxylase) (Extended Data Fig. 3b).

### Proteomic fingerprinting of LSD mutant iN and iDA neurons

**Fig. 2:**
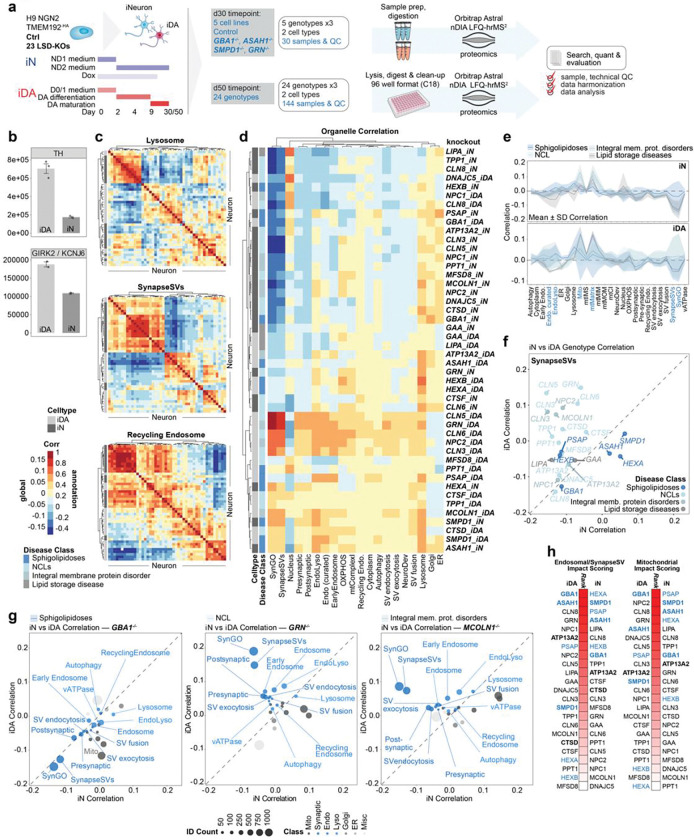
Proteome atlas of lysosomal Storage Disorder neuronal cell models. **a**, Schematic of experimental workflow. Control and 23 LSD knockout human ES cell lines were differentiated to iN and iDA in two cohorts (day 30, day 50, each in biological triplicate) and analyzed using nDIA LFQ. **b**, Example abundance of neuronal markers in Control iDA and iN at day 50 of differentiation. **c**, Example of pairwise, proteome-wide cross-correlation of lysosome (top), SynapseSV (middle) and recycling endosome annotated proteins. **d**, Composite correlation across 20 organelle annotations. **e**, Mean organelle correlation based on by disease classes. ± SD for iN (top) and iDA (bottom). Disease class is indicated by shading. **f**, iN vs. iDA organelle correlation of SynapseSV proteins. Genotypes are color-coded by disease class. **g**, iN vs. iDA organelle correlation plots for *GBA1*^−/−^ (left), *GRN*^−/−^ (middle) and *MCOLN1*^−/−^ (right). **h**, Ranked impact scoring based on SynapseSV and vesicular annotations (endosomes, etc; left) and mitochondrial annotations (right).

To profile alterations in the proteome of our LSD mutant collection, we created day 50 iN and iDA cells in biological triplicate for 23 mutant lines along with parental Control cells (Fig. 2a; **Supplementary Table 4**). In parallel, a focused set of cell lines lacking genes linked with sphingolipidoses (*ASAH1*, *GBA1*, *SMPD1*, as well as *GRN*) were differentiated for 30 days, in biological triplicates (Fig. 2a; **Supplementary Table 5**). Based on previous reports, these time points are sufficient to observe synaptic activity^26,27^. Total cell proteins and interspersed quality control samples were subjected to analysis using an Astral mass spectrometer with nDIA-hrMS2 (narrow-window data-independent acquisition-high resolution MS^2^) acquisition (Fig. 2a). We routinely detected ~10,000 proteins across samples, with consistent instrument performance and relative standard deviation <10% (Extended Data Fig. 3c-e; **Supplementary Table 4 and 5**). As expected, TH was elevated in iDA cells as compared with iN cells (Fig. 2b). Moreover, many proteins associated with the synaptic compartment were increased in abundance in Control cells during the day 30 to day 50 interval, consistent with continued synaptic maturation (Extended Data Fig. 3f,g). The expected loss of the targeted protein was also reduced or absent, when detected (Extended Data Fig. 3h).

To elucidate alterations within the proteomes of LSD mutants across iN and iDA cell types at day 50, we calculated pairwise correlations of all genotypes across groups of proteins with specific sub-cellular annotations (Fig. 2c,d), including both general organellar terms (e.g. nucleus, Golgi, endoplasmic reticulum (ER)) and more focused groups of partially overlapping annotations within the synaptic, SynGO, endolysosomal and mitochondrial compartments (Extended Data Fig. 3i, see METHODS). Subcellular organelle annotations displayed varying correlation clusters, for example, with lysosome, SynapseSV, and recycling endosome, across the dataset (Fig. 2c). To capture the global impact of each lysosomal gene knockout, we generated an organelle correlation map by computing all pairwise correlations of annotation-specific mean log_2_fold-changes across genotypes and cell types. This composite map integrates the direction and strength of these relationships (as a correlation factor) to summarize how each knockout reshapes proteome organization across subcellular compartments. By comparing annotations against the entire dataset, the map highlights those organelles that undergo coordinated abundance shifts, pointing towards compartments that are particularly sensitive to lysosomal dysfunction. This systems-level perspective enables the identification of organelle clusters that co-vary in response to gene loss, revealing higher-order patterns of cellular remodeling that might underlie disease-relevant phenotypes (Fig. 2d). For example, both *CLN5*^−/−^ and *GRN*^−/−^ iDA share high similarity in synaptic annotation categories (SynGO, SynapaseSV), whereas iN of *SMPD1*^−/−^ and *ASAH1*^−/−^ clustered together with iDA of *SMPD1*^−/−^ and *CTSD*^−/−^ (but interestingly not ASAH1^−/−^ iDA). When comparing alterations in iNeuron proteomes with the corresponding LSD gene deletion set in HeLa cells reported previously, we could generally stratify the correlations into three groups: 1) high positive correlations, as seen with mitochondria, 2) intermediate positive correlations, as seen with early endosomal proteins, and 3) negative correlation with synapse protein annotations, as would be expected when comparing iNeurons versus HeLa cells (Extended Data Fig. 3j). Thus, a subset of organellar proteome alterations with deletion of specific LSD genes are found across diverse cell types.

**Extended Data Fig. 4:**
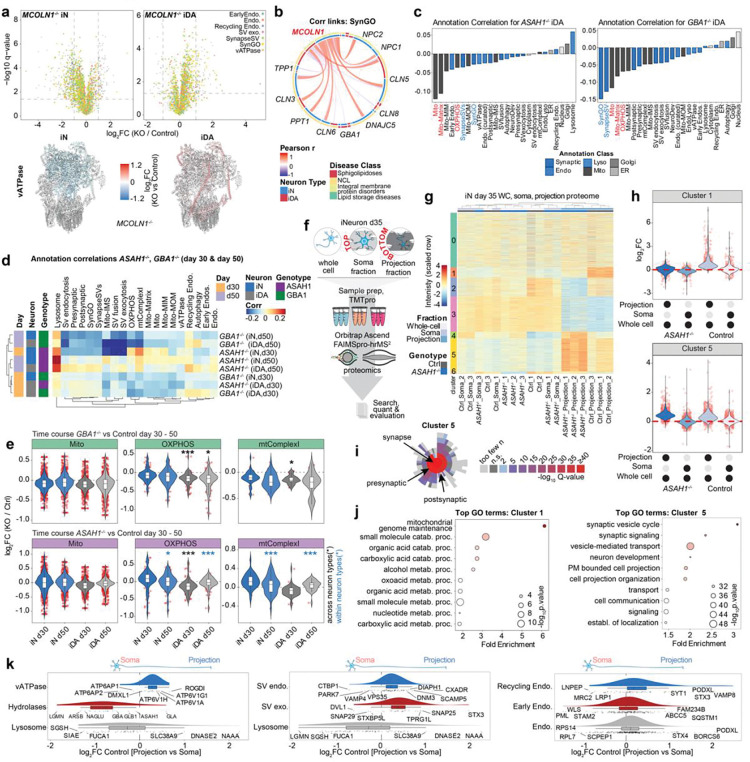
Proteomic analysis of *ASAH1*^−/−^, *GBA1*^−/−^ and *MCOLN1*^−/−^ induced neurons and *ASAH1*^−/−^ neuronal projections. **a**, Top, Volcano plot [log_2_FC (*MCOLN1*^−/−^ /Control) versus −log_10_ q-value] for iN (left panel) and iDA (right panel) cells. Various cellular compartments are annotated. Bottom, Log_2_FC of vATPase subunits for iN (left) and iDA (right) are mapped onto the cryo-EM structure (PDB: 6WM2)^39^. **b**, Circos plot of selected annotation correlation (here: SynGO) links between *MCOLN1*^−/−^ and proteomes from other LSD mutant cell lines. **c**, Ranked barplots depicting correlation of indicated annotations of select genotypes vs the remaining 22 LSD mutant cell lines. Left plots shows on *ASAH1*^−/−^ iDA proteome, right plot shows *GBA1*^−/−^ iDA proteome. **d**, Heatmap depicting correlations between organelle annotations (columns) and *GBA1*^−/−^ or *ASAH1*^−/−^ iN or iDA cells (rows) for both day 30 and day 50 *in vitro* differentiation timepoints. **e**, Violin plots depicting log_2_FC of mitochondrial, OXPHOS and mtComplexI annotations (from left to right) for *GBA1*^−/−^ (top) or *ASAH1*^−/−^ (bottom) iN or iDA cells across day 30 and day 50 *in vitro* differentiation timepoints. **f**, Schematic diagram of experimental workflow for quantitative assessment of whole-cell, neuronal somata, and neuronal projections. **g**, Heatmap of TMTpro-based proteomics of day 35 iN *ASAH1*^−/−^ and Control whole-cell, soma and neuronal projections. Cluster designations are labeled on the left. **h**, Violin plots of clusters 1 and 5 depicting log_2_FC for either whole cell vs projection or whole cell vs soma fraction for both *ASAH1*^−/−^ and Control iN cells. **i**, SynGO localization analysis of protein IDs belonging to cluster 5. **j**, Top 10 GO enrichment for cluster 1 (left) and cluster 5 (right). **k**, Rainfall plot of log_2_FC (projections vs soma) of day 35 *in vitro* differentiated Control iN cells, depicting distribution of vATPase, lysosomal hydrolases and lysosomal proteins (left) and SV endocytosis, SV exocytosis and lysosomes (middle) or recycling endosomal, early endosomal and endosomal proteins (right).

To understand the extent of proteome diversity across individual classes of LSD mutant cell lines, we examined proteome variance within disease classes across sub-cellular/organelle localizations (Fig. 2e). The highest variability in correlations was found for groups of proteins linked with mitochondria, endolysosome, and synaptic vesicles/SynGO, for both iN and iDA neurons (Fig. 2e). For example, the SynapseSV category of proteins displays high variance across genotypes, particularly in iDA neurons, while with iN’s, the sphingolipidoses group of proteins displayed the most extensive variability (Fig. 2e,f). Distinct patterns of variation between organelles can be visualized in correlation plots for iN versus iDA neurons with particular LSD mutations (Fig. 2g). For example, synaptic proteins are among the most variable (most negative correlations, compared to rest of dataset) in both iDA and iN *GBA1*^−/−^ (Sphingolipidoses) cells, while mitochondria are selectively affected (negatively correlated) in iDA cells (Fig. 2g). In contrast, synaptic proteins were positively correlated in iDA *GRN*^−/−^ (NCL) or *MCOLN1*^−/−^ cells, but were negatively correlated in iN cells (Fig. 2g). Moreover, subunits of vATPase, a component of both synaptic vesicles and the endolysosomal system, were decreased in *MCOLN1*^−/−^ iN cells but increased in *MCOLN1*^−/−^ iDA cells (Extended Data Fig. 4a). Interestingly, alterations in synaptic proteins represented in SynGO in *MCOLN1*^−/−^ cells displayed strong positive correlations with *CLN5*^−/−^ and *CLN3*^−/−^ mutants in both iDA and iN lineages, while *MCOLN1*^−/−^ iN cells selectively showed positive correlations with *NPC1*^−/−^, *NPC2*^−/−^, *DNAJC5*^−/−^, *CLN6*^−/−^, *PPT1*^−/−^ and *TPP1*^−/−^ iN cells (Extended Data Fig. 4b).

As an orthogonal approach to comparing the effects of LSD mutations, we implemented a ranked impact score that assesses the extent of variability in endosomal/synaptic vesicle or mitochondrial proteins in either iDA or iN cells (Fig. 2h) (see METHODS). In iDA cells, mutations linked with Sphingolipidoses were distributed across the rank order, with *GBA1*^−/−^ and *ASAH1*^−/−^ cells showing high impact scores for both endosomal/synaptic vesicle and mitochondrial compartments, while *HEXA*^−/−^ and *HEXB*^−/−^ cells displayed low impact scores. By contrast, *HEXA*^−/−^ and *HEXB*^−/−^ largely clustered with *GBA1*^−/−^ and *ASAH1*^−/−^ in iN cells across both compartments (Fig. 2h). This analysis suggests possible differences in cellular vulnerabilities in iDA versus iN cells with distinct LSD mutations.

### Decoding Mitochondrial Proteome Signatures within Sphingolipidoses-related LSD neuronal models

Sphingolipidoses genes in our dataset include *GBA1*, *ASAH1*, *SMPD1*, *PSAP*, *HEXA*, and *HEXB*, all of which are involved in the breakdown of sphingolipids (Extended Data Fig. 1a, 3a). Among these, *GBA1*, *ASAH1*, and *SMPD1* are associated with an increased incidence of PD, with *GBA1* being a particularly strong risk factor^10,12,13,15,20^. *GBA* and *ASAH1* mutants displayed a much stronger impact score ranking than did, for example, *HEXA* and *HEXB* mutants in iDA cells for both endolysosomal/synaptic and mitochondrial compartments, two compartments known to be vulnerable in PD^28–31^ (Fig. 2h) led us to further assess the impact of these mutations on proteome alterations within the Sphingolipidoses class of LSDs (referred to as SphMut). For this analysis, we also included *GRN*^−/−^ mutants, as loss of *GRN* is associated with defects in sphingolipid metabolism due to reduced BMP levels^7,32^.

**Fig. 3:**
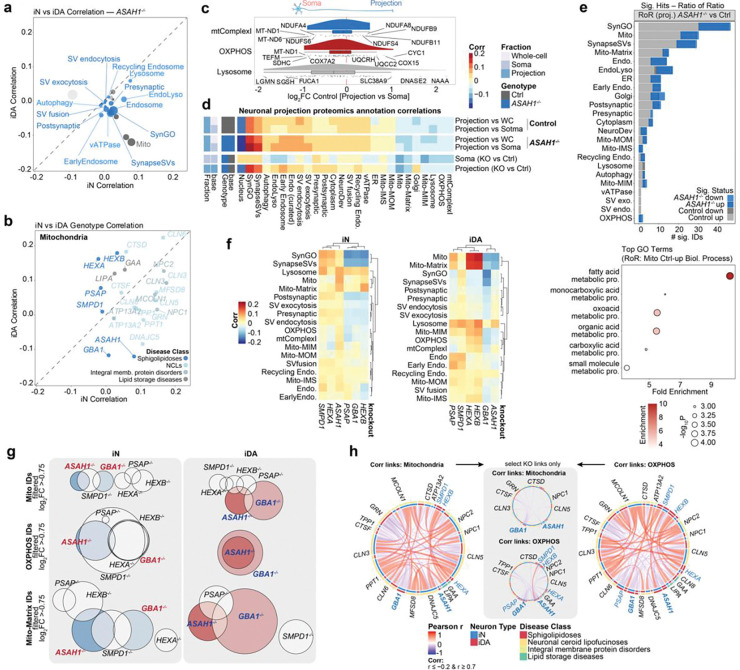
Mitochondrial defects are manifested in iDA neurons lacking *ASAH1.* **a**, iN vs. iDA organelle correlation plots for *ASAH1*^−/−^ cells. **b**, iN vs. iDA organelle correlation of mitochondrial proteins across mutant cell lines. Genotypes are color-coded by disease class. **c**, Rainfall plot of log_2_FC (projections vs soma) of day 35 *in vitro* differentiated Control iNeurons, depicting distribution of proteins belonging to mtComplexI, OXPHOS, and lysosome annotations. **d**, Heatmap of correlation for indicated annotations of day 35 iN *ASAH1*^−/−^ and Control whole-cell, soma and neuronal projections. Data based on TMTpro-based proteomics in biological triplicates. **e**, Top: stacked bar graph depicting the number of significant hits (up vs down) for both RoR-normalized (Ratio of Ratio; *ASAH1*^−/−^ $\left(\frac{projection}{whole\;cell}\right)/\text{Control}\;(\frac{projection}{whole\;cell})$) *ASAH1*^−/−^ and Control neuronal projections. Bottom: Example GO enrichment (biological process) of significant hit for Control iN cells. **f**, Correlation heatmap of indicated organelle annotations across sphingolipidoses mutants for iN (left) and iDA (right) cells. **g**, Euler plots depicting overlap and unions of mitochondrial (top), OXPHOS (middle) and mito-matrix (bottom) protein IDs log_2_FC > −0.75 across sphingolipidoses mutants for iN and iDA cells. **h**, Circos plots showing correlations between genotypes for mitochondria (left) and OXPHOS (right). Center panel depicts genotypic links originating from only *GBA1*^−/−^ and *ASAH1*^−/−^. Only links with Pearson scores (r) smaller than −0.2 and larger than 0.7 are shown. Blue labels indicate genotypes belonging to Sphingolipidoses disease class. Bold labels are PD-risk genes.

As with *GBA1*^−/−^ mutants, day 50 iN versus iDA correlation plots for *ASAH1*^−/−^ cells identified mitochondria as anti-correlated in the two cell types (Fig. 2g and 3a, Extended Data Fig. 4c). Indeed, iDA and iN correlation plots for the mitochondrial compartment revealed that *GBA1*^−/−^ and *ASAH1*^−/−^ mutants appear distinct from *SMPD1*^−/−^, *PSAP*^−/−^, *HEXA*^−/−^, and *HEXB*^−/−^ mutants, placing *GBA1*^−/−^ and *ASAH1*^−/−^ in an apparently unique category (Fig. 3b).

We wondered if not only the abundance but also the distribution of mitochondrial proteins is affected by the loss of *ASAH1*. We performed Tandem Mass Tagging (TMT)-based 18-plex proteomic analysis of triplicate whole cell, soma and projection fractions from Control and *ASAH1*^−/−^ iN cells (day 35) grown in Transwell dishes (Extended Data Fig. 4f; **Supplementary Table 6**). Cluster analysis demonstrated synaptic enrichment of the neuronal projection fractions (Cluster 5, Extended Data Fig. 4g-j), suggesting successful separation of the different cellular compartments. Mitochondrial proteins were enriched towards the soma of Control cells, as expected (Fig. 3c). Moreover, cluster analysis (Extended Data Fig. 4g-j) revealed increased abundance of a set of proteins (Cluster 1) in projections from Control but not knockout cells, which are enriched in mitochondria genome maintenance GO terms (Extended Data Fig. 4h,j). Likewise, mitochondrial compartments were the most negatively correlated when comparing *ASAH1*^−/−^ and Control cells in either the soma or projection compartments (Fig. 3d). Given that all samples were part of the same TMT-plex, we performed a Ratio-of-Ratio (RoR) evaluation, where the neuronal projection fractions are normalized to the whole-cell fraction of the same genotype, allowing a determination of significantly altered proteins on a compartment basis (Fig. 3e). Consistent the cluster evaluation, mitochondrial and synaptic proteins were relatively increased in neuronal projections, especially in Control cells. mtMatrix, synaptic proteins and proteins involved in fatty acid metabolism were underrepresented in *ASAH1*^−/−^ projections when compared to Control cells (Fig. 3e). Unsupervised hierarchical clustering of individual mitochondrial, endosomal, and synaptic correlations in iN cells across the SphMut dataset revealed *ASAH1*^−/−^, *SMPD1*^−/−^, and *HEXA*^−/−^ cells to be most similar to each other (Fig. 3f).

In this context, *ASAH1*^−/−^ iNs displayed a distinct pattern in the mito and mito-matrix sub-organelle categories from *GBA1*^−/−^, which was reversed in iDA cells (Fig. 3f). Indeed, in contrast to iN cells, *GBA1*^−/−^ and *ASAH1*^−/−^ mutants in iDA cells were most similar to each other in unsupervised organelle correlations. This effect appeared to reflect a switch from positive to negative correlations for mitochondrial and synaptic proteomes in *ASAH1*^−/−^ mutants when comparing iN and iDA cells from the SphMut genotypes (Fig. 3f). This was especially intriguing, since mitochondrial dysfunction in DA neurons has been previously reported to induce Parkinsonism in mouse models^23^. Follow-up evaluation of day 30 and day 50 proteomes-subsets of *ASAH1*^−/−^ and *GBA1*^−/−^ cells revealed a significant reduction in OXPHOS and mtComplexI proteins especially in iDA neurons of *ASAH1*^−/−^, mirroring the *GBA1*^−/−^ phenotype (Extended Data Fig. 4d,e). We separated specific mitochondrial proteins based on functional categories and abundance, including proteins involved in oxidative phosphorylation (OXPHOS) and mitochondrial matrix (mito-matrix), and examined overlap between the various SphMut genotypes in iN and iDA cells (Fig. 3g). While *GBA1*^−/−^ and *ASAH1*^−/−^ mutants displayed either complete (OXPHOS) or partial (mito- and mito-matrix) overlap in proteins with altered abundance in iDA cells, no overlap was observed in the context of iN cells (Fig. 3g). Consistent with OXPHOS components underlying the dependency on neuron subtype, negative correlation connectivity (links) with other LSD gene deletions were prominent for *ASAH1*^−/−^ iDA cells in the context of OXPHOS proteins, but were much less prominent in the context of total mitochondrial proteins (Fig. 3h). Interestingly, the only positive link observable in the dataset for *ASAH1*^−/−^ cells was with *SMPD1*^−/−^, and this was specific to iN cells (Fig. 3h). Moreover, in contrast with mitochondrial proteins, synaptic proteins were positively correlated most strongly in *ASAH1*^−/−^ projections relative to either soma or to projections from Control iN cells in the spatial proteomics analysis introduced above (Fig. 3e). Cluster 5 in this analysis (Extended Data Fig. 4g,h) was enriched in several classes of synaptic proteins based on GO terms whose abundance is increased in projections from *ASAH1*^−/−^ iN cells, relative to control cells (Extended Data Fig. 4i,j). As expected, lysosomes and its resident hydrolases were enriched in the soma, while vATPase, SV-endocytosis, and SV-exocytosis were enriched in projections (Extended Data Fig. 4k). Proteins associated with recycling endosome terms were slightly enriched in projections relative to soma in control cells, with SNARE proteins VAMP8 and STX3, the synaptic vesicle fusion protein SYT1, and the candidate recycling cargo protein PODXL being particularly enriched (Extended Data Fig. 4k). Together, these data provide insight into how loss of specific SphMut genes alters the abundance of proteins within the mitochondrial compartment in a neuronal type-dependent manner.

### Decoding Synaptic and Endolysosomal Proteome Signatures within Sphingolipidoses-related LSD neuronal models

**Fig 4:**
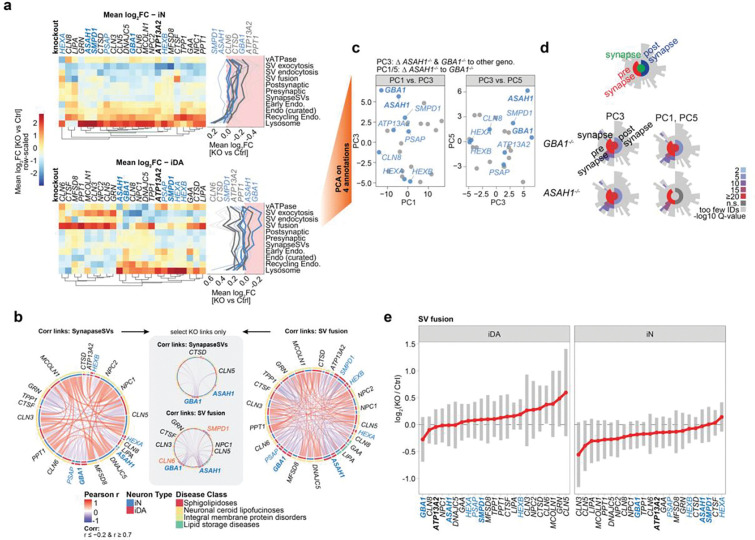
Systematic analysis of synaptic and endolysosomal compartments in sphingolipidoses mutant induced neurons. **a**, Log_2_FC [KO vs Control] heatmap of vesicular annotations for iN (top) and iDA (bottom) cells at day 50 of differentiation. The annotations are sorted from high to low from left to right based on iN cell data. Line plots (right) show abundance traces of select genotypes of interest (labeled). Sphingolipidoses related genotypes are in blue and LSD-PD risk genes are labeled in bold. **b**, Circos plots showing correlations between genotypes for SynapseSVs (left) and SV fusion (right). Center plots depict correlation links originating from only *GBA1*^−/−^ and *ASAH1*^−/−^ . Only links with Pearson (r) smaller than −0.2 and larger than 0.7 are shown. Blue labels indicate genotypes belonging to Sphingolipidoses-risk genes. Bold labels are PD-risk genes. **c**, Example PCA plots of top 4 annotations, highlighting PC1, 3 and 5 as explaining main differences between *GBA1*^−/−^ and/or *ASAH1*^−/−^ and other Sph-mutants (PC3) and between the two genotypes (PC1 and PC5). **d**, Sunflower plots of SynGO enrichment for the components of the indicated PCA analyses from panel **b**. **e**, Ranked log_2_FC (KO/Control) abundance for SV fusion annotated proteins across indicated genotypes for iDA (left) and iN (right).

We next performed an analogous analysis of synaptic and endolysosomal compartments in both iN and iDA cells, which appeared to be important drivers of distinct patterns of correlations across SphMut cells (Fig. 3f). This analysis revealed a frequent, although not universal, increase in the abundance of endolysosomal proteins across iN LSD genotypes (Fig. 4a). Alterations in abundance were particularly striking in recycling endosome and lysosomal annotations. By contrast, in iDA cells, two major patterns were observed: a subset of LSD mutants, including all SphMut genes, which like iN cells displayed an increased in the abundance of endolysosomal proteins, and a second set of knockout cell lines, including several NCL genes, which displaying decreased expression (Fig. 4a, lower panel). Interestingly, this pattern was partially mirrored in iN cells when view through the lens of SV-fusion, SV-exocytosis, and SV-endocytosis proteins, which typically displayed reduced abundance (Fig. 4a). However, iDA cells displayed a more complex picture, with a subset of LSD genotypes, including SphMut cells, displaying reduced SV-exocytosis and SV-endocytosis protein levels, largely mirroring the increased abundance of endolysosomal proteins (Fig. 4a). Most SphMut cells displayed reductions in SV-endocytosis and SV-exocytosis protein abundance, but unlike other genotypes, *GBA1*^−/−^ and *ASAH1*^−/−^ cells also showed reductions in the abundance of SV-fusion proteins in iDA neurons (Fig. 4a). *ASAH1*^−/−^ mutants also displayed a modest reduction in vATPase subunit abundance (Fig. 4a). Proteins within the SV-fusion category displayed numerous negatively correlated links with specific LSD mutants in either iDA or iN cells, and as expected, the number of such links within the SynapseSV category was substantially reduced relative to SV-fusion (Fig. 4b). Alterations in synaptic proteins are also evident in PCA analysis, where *GBA1*^−/−^ and *ASAH1*^−/−^ genotypes separated from the other groups in PC3 and from each other in comparing PC3 with PC5 (Fig. 4c,d). Primary drivers of the effects seen in *ASAH1*^−/−^ cells include several calcium binding proteins that are involved in synaptic vesicle fusion in the pre-synapse, including DOC2B, SYT1, SYT2 and SYT7, as well as the absence of specific synaptic proteins in comparing PC1 versus PC5 for the two genotypes (Fig. 4c). We then examined the ranked abundance of SV-fusion proteins in either iDA or iN cells across the LSD mutant in our collection. SV-fusion proteins in *GBA1*^−/−^ and *ASAH1*^−/−^ cells ranked among the lowest in iDA cells but not iN cells (Fig. 4e).

**Extended Data Fig. 5:**
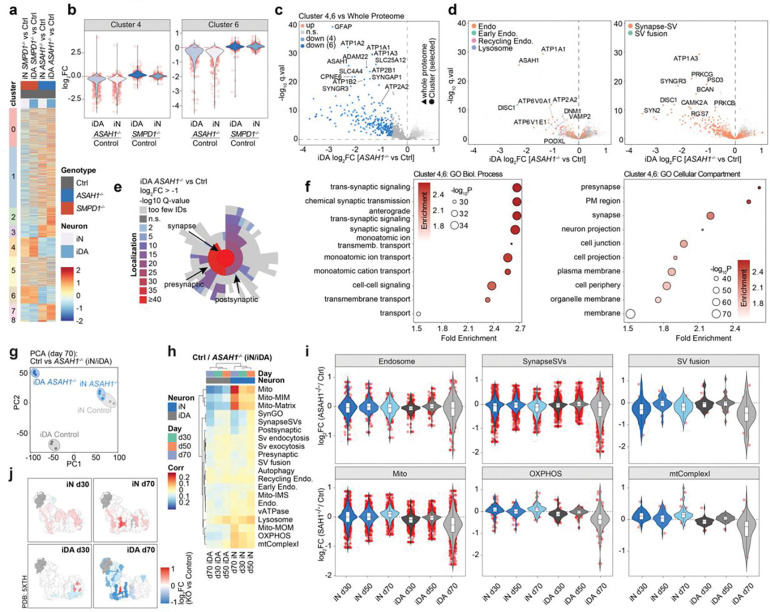
Loss of *ASAH1*^−/−^ disturbs synaptic and mitochondrial proteome pools and endo-lysosomal morphology. **a**, Unsupervised hierarchical clustering heatmap of log_2_FC *ASAH1*^−/−^ or *SMPD1*^−/−^ vs Control iN or iDA cells. Clusters designations are provided on the left. **b**, Violin plots (log_2_FC) of proteins in cluster 4 and 6 in *ASAH1*^−/−^ and *SMPD1*^−/−^ iN or iDA cells. **c**, Volcano plot [log_2_FC (*ASAH1*^−/−^ /Control) vs. −log_10_ q-value] iDA cells at day 23 of *in vitro* differentiation. Significantly down regulated protein IDs in cluster 4 are highlighted in light blue; proteins from cluster 6 in dark blue. Whole proteome is represented in triangles, clusters of interest in dots. **d**, As in panel c, but depicting proteins linked with endolysosomal compartments highlighted in the indicated colors against the grey background for whole-cell proteome. **e**, SynGO localization plot of protein IDs log_2_FC > −1 *ASAH1*^−/−^ vs Control in iDA cells. **f**, GO enrichment of cluster 4 & 6 for biological process (left) and cellular component (right). Top 10 hits are shown. **g**, PCA plot for LFQ-nDIA proteomics of Control and *ASAH1*^−/−^ iN and iDA cells at day 70 of *in vitro* differentiation. **h**, Correlation heatmap of annotations across day 30, 50 and 70 of *in vitro* differentiation for both iN and iDA neurons. Data based on (*ASAH1*^−/−^ vs Control) whole cell LFQ-nDIA proteomics. **i**, Violin plots depicting log_2_FC abundance [*ASAH1*^−/−^ vs Control] across day 30, 50 and 70 of *in vitro* differentiation for endosome, SynapseSVs and SV fusion annotations (top row; from left to right) or mitochondria, OXPHOS and mtComplexI annotation (bottom row). **j**, Log_2_FC abundance [*ASAH1*^−/−^ vs Control] of mtComplexI for iN (top) and iDA (bottom) cells at day 30 and 70 of *in vitro* differentiation are mapped onto the cryo-EM structure (PDB: 5XTH)^40^.

Alterations in the synaptic compartment in *ASAH1*^−/−^ iDA and iN cells could also be observed as early as day 23 of differentiation, as determined by TMT-based analysis of Control, *ASAH1*^−/−^ and *SMPD1*^−/−^ cells (Extended Data Fig. 5a-f; Supplementary Table 7). This includes two clusters (4 and 6) that are enriched in GO biological process or cellular compartment terms related to synaptic function (Extended Data Fig. 4f), and enrichment of pre- and post-synaptic SynGO terms (Extended Data Fig. 5e). Taken together, these data indicate that selective alterations in specific components of endolysosomal, mitochondrial and synaptic proteome in *GBA1*^−/−^ and *ASAH1*^−/−^ mutants, especially in iDA cells.

### Synaptic Defects in *ASAH1*^−/−^ iDA cells

The continued maturation of iN and iDA neurons based on further accumulation of synaptic proteins from day 30 to day 50 led us to examine further alterations in the proteomes of Control and *ASAH1*^−/−^ cells at day 70 of differentiation (**Supplementary Table 8**). While Control and *ASAH1*^−/−^ day 70 iN cells displayed closely related proteomes based on PCA analysis, Control and *ASAH1*^−/−^ iDA cells are separated both from iN cells (PC1) and from each other (PC2) (Extended Data Fig. 5g). Interestingly, iN cells displayed positive correlations for mitochondrial organelle categories across the 30 to 70 day time course, while iDA cells displayed negative correlations (Extended Data Fig. 5h), suggesting distinct behavior based on their neuronal identity. Indeed, day 70 iDA cells displayed reduced levels of numerous mitochondrial proteins (log_2_FC *ASAH1*^−/−^/Control) that was not observed in iN cells (Extended Data Fig. 5i). This includes several proteins in the membrane module of mtComplex I as well as the matrix Q-module (Extended Data Fig. 5i,j). In contrast with mitochondria and SV-fusion proteins, the abundance of endosomal proteins were maintained in day 70 iDA cells, indicating that not all organelles are vulnerable to time-dependent loss in expression (Extended Data Fig. 5i).

**Extended Data Fig. 6:**
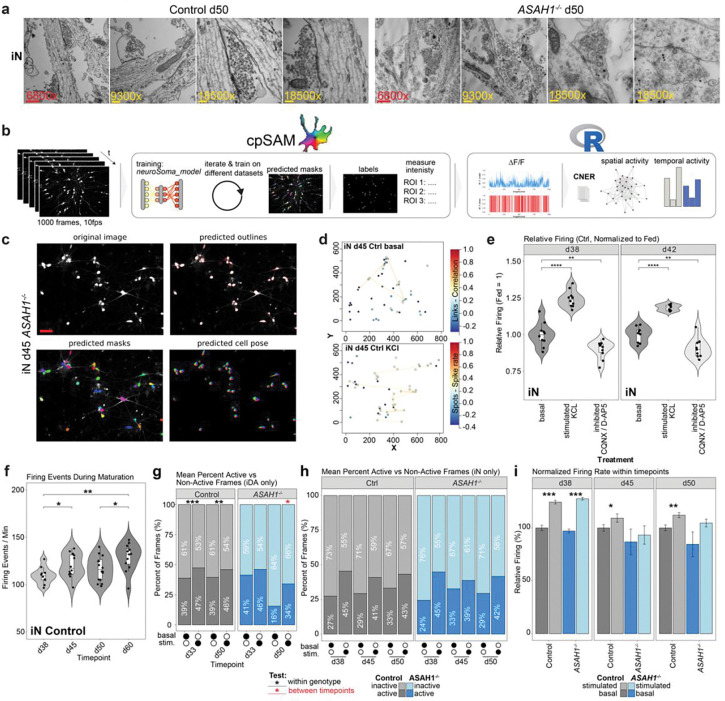
Analysis of synaptic function in *ASAH1*^−/−^ iN cells. **a**, Representative EM images depicting synapses and neuronal ultrastructure of Control and *ASAH1*^−/−^ iN cells at day 50 of *in vitro* differentiation. **b**, Schematic of Machine Language-assisted neuronal calcium evaluation pipeline. nd2 time-series are segmented using custom-trained cellposeSAM (cpSAM) models. Calcium intensities over time were then analyzed in R. **c**, Stills of live-cell spinning-disk confocal images of day 45 iN cells (original image), predicted outlines and masks as well labels from cellposeSAM. Scale bar: 50 μm. **d**, Example activity network of d45 *ASAH1*^−/−^ iN cells under basal (top) and KCl stimulated (bottom) conditions. Dots are color-coded by individual spike rate and links between dots depict activity correlations (synchronicity). **e**, Violin plots of relative firing rate of day 38 and day 42 Control iN cells under basal, stimulated and inhibited conditions. d38: **P(0.006); ****P(0.0001); d42: **P(0.01); ****P(0.0002). **f**, Basal neuronal activity in Control iN cells over the length of maturation time. d38: *P(0.05); **P(0.008); d50 *P(0.05). **g**, Stacked bar graph for Control (left) and *ASAH1*^−/−^ (right) iDA cells depicting the mean % of active and non-active frames (ROIs) for any given time. Data from day 33 and 50 of *in vitro* differentiation with or without stimulation by KCl. Control d33 fed vs KCL***P(0.0001); d50 fed vs KCL:**P(0.007); *ASAH1*^−/−^ d33: fed vs KCL***P(0.00006); d33 vs 50 KCL: *P(0.03). **h**, Stacked bar graph for Control (left) and *ASAH1*^−/−^ (right) iN cells depicting the raw mean % of active ROIs at any given frame. Data from day 38, 42 and 50 of *in vitro* differentiation. **i**, Bar plot of normalized firing rate in iNeurons for Control and *ASAH1*^−/−^ iN cells for day 38, 42 and 50 of *in vitro* differentiation. Values are normalized to Control basal within each timepoint. d38: ***P(0.0001); d45: *P(0.06); d50: **P(0.01).

**Fig. 5:**
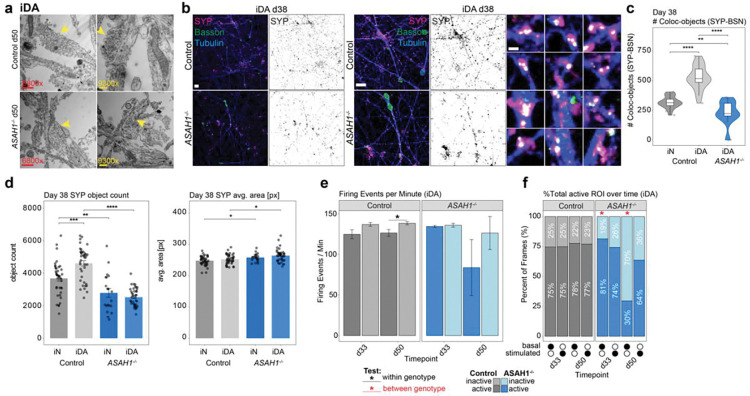
Synaptic morphology defects correlate with reduced basal Ca^2+^ firing activity in *ASAH1*^−/−^ iDA cells. **a**, Representative EM images depicting synapses and neuronal ultrastructure of Control and *ASAH1*^−/−^ iDA neurons at day 50 of *in vitro* differentiation. Scale bars are indicated. Pre-synaptic structures with synaptic vesicles are indicated by yellow arrowheads. **b**, Confocal images of Control and *ASAH1*^−/−^ iN and iDA cells at day 38 of *in vitro* differentiation, labeled for SYP, Bassoon and Tubulin. Scale bar: 20 μm and 2 μm. **c**, Number of colocalized SYP-Bassoon object pairs in Control iN and iDA and iDA *ASAH1*^−/−^ neurons, based on confocal images in panel **b** (n(Control iN): 20 stacks; n(Control iDA): 19 stacks; n(*ASAH1*^−/−^ iDA): 19 stacks). iN: **P(0.001); iDA: ****P< 0.0001; iDA Ctrl vs *ASAH1*^−/−^ : ****P< 0.0001. **d**, Quantification of SYP object counts and area (from left to right), based on confocal images in panel **b**. (n(Control iN): 40 stacks; n(Control iDA): 40 stacks; n(*ASAH1*^−/−^ iN): 20 stacks; n(*ASAH1*^−/−^ iDA): 40 stacks). Object count: Control iN vs iDA: ***P(0.0002), iN Control vs *ASAH1*^−/−^ : **P(0.003), iDA Control vs *ASAH1*^−/−^ : ****P< 0.0001; Object area: *P(0.01). **e**, Bar graph of firing events per minute of Control and *ASAH1*^−/−^ iDA cells at day 33 and 50 of *in vitro* differentiation. Control d50 fed vs KCL:*P(0.05). **f**, Stacked bar graph for Control (left) and *ASAH1*^−/−^ (right) iDA cells depicting the cumulative % of active ROIs over the time course. d33 Control vs *ASAH1*^−/−^ : *P(0.02); d50 Control vs *ASAH1*^−/−^ : *P(0.04). Data from day 33 and 50 of *in vitro* differentiation with or without stimulation by KCl.

The results of our proteomics analysis of *ASAH1*^−/−^ cells led us to examine possible alterations in synaptic ultrastructure. We first performed thin-section electron microscopy on day 50 Control and *ASAH1*^−/−^ iDA and iN cells (Fig. 5a, Extended Data Fig. 6a). In Control iDA cells, we observed pre-synaptic structures containing numerous synaptic vesicles with uniform vesicle sizes (Fig. 5a). In contrast, apparent synaptic structures in *ASAH1*^−/−^ iDA cells appeared more disorganized with often fewer synaptic vesicles and with less uniform size distributions (Fig. 5a). Similar findings were made in day 50 iN cells (Extended Data Fig. 6a). Immunostaining of day 38 iDA cultures with the pre-synaptic markers SYP (synaptophysin) and BSN (Bassoon) revealed largely uniform pre-synaptic with the majority of SYP puncta co-staining with Bassoon in Control cells (Fig. 5b-d). In contrast, *ASAH1*^−/−^ iDA cells frequently displayed enlarged α-tubulin positive neuronal structures that contained α-SYP puncta that was more frequently not co-stained with α-Bassoon (Fig. 5b-d), suggesting defects in pre-synaptic compartment organization.

To examine the functional impact of *ASAH1* deficiency, we performed Fluo-4-based Ca^2+^ imaging of Control and *ASAH1*^−/−^ iDA and iN cells over increasing time periods of differentiation. To facilitate quantification of neuronal firing rates, we implemented a robust evaluation pipeline that allows for the segmentation of soma using the machine-learning tool cellposeSAM^33^ based on custom-trained segmentation models, linked with CalciumNetExploreR (CNER), a calcium analysis package in R^34^ (Extended Data Fig. 6b-e). We observed robust segmentation of neuronal soma (Extended Data Fig. 6c), as well as an increased relative firing frequency after stimulation with KCL, which could be reduced by pre-treatment with CQNX/D-AP5 (Extended Data Fig. 6d,e). Firing rates in iN cells also increased with time of differentiation from day 38 to day 45 or day 60 (Extended Data Fig. 6f). We found that basal firing rates in day 50 *ASAH1*^−/−^ iDA cells were reduced, as compared with Control cells, but this reduction was largely reversed in the presence of KCl (Fig. 5e). The percent of total active regions of interest (ROI) over time and the mean percentage of active versus non-active frames were likewise significantly reduced in day 50 iDA *ASAH1*^−/−^ vs Control cells, but this reduced activity could not be rescued to Control-levels by the addition of KCl (Fig. 5f, Extended Data Fig. 6g). Additionally, when comparing activity across timepoints (d33, d50), only *ASAH1*^−/−^ iDA cells demonstrated a significant reduction in activity. In contrast, firing rates were only minorly affected in iN cells over the selected timepoints (Extended Data Fig. 6h,i). Thus, alterations in the synaptic proteome in iDA cells correlates with reduced firing rates at day 50 of differentiation.

## DISCUSSION

Here we report the creation of an ES cell toolkit for analysis of 23 LSD genes in differentiated neuronal states. This toolkit provides a means by which to examine functional consequences of individual LSD gene loss during changes in cell states while also allowing comparisons across various classes of storage disorders. Through analysis of total proteomes in both iN and iDA cells, coupled with correlation analysis of proteins that reside in specific organelles or functional pathways, we provided a landscape of alterations reflecting the activities of individual LSD genes. Interestingly, iDA cells and iN cells displayed distinct patterns of organelle proteome remodeling. To our knowledge, this is the first comparison between iN and iDA cells in culture at this level of proteome depth, and the first comparison across divergent LSD mutants.

Our analysis focused on genes linked with sphingolipidoses, due to potential links between *GBA1*, *ASAH1*, and *SMPD1* in PD risk^20^. We found that *GBA1* and *ASAH1* mutants behaved in a distinct manner from other sphingolipidoses genes in terms of the patterns of proteome alterations observed in iN and iDA cells. In particular, we observed alternations in OXPHOS and pre-synaptic proteins in *GBA1* and *ASAH1* mutant iDA cells. The selectivity observed for alterations in the mitochondrial OXPHOS system in iDA cells is interesting in light of the known linkage between OXPHOS pathways and PD^22,23^. Conditional deletion of murine mtComplex I subunit *Ndufs4* results in a deficit in nigrostriatal axons, and is associated with a PD-like loss of motor function, suggesting that loss of mtComplex I component of OXPHOS is sufficient to produce motor dysfunction^23^. Likewise, the mtComplex I inhibitors MPTP and rotenone promote a Parkinson’s like phenotype in mice^35,36^. Similarly, synaptic dysfunction has also been linked with PD, including synaptotagmin-11 (*SYT11*) and synaptojanin (*SYNJ1*) variants that have been associated with familial PD^37,38^. It is conceivable that alterations in OXPHOS and pre-synaptic protein pools observed in *ASAH1*^−/−^ and *GBA1*^−/−^ iDA cells could represent vulnerabilities that increase the risk of PD in patients with reduced enzymatic activity for these key sphingolipid catabolism enzymes. It is also possible that reduced activity of mitochondria within the synaptic compartment contributes to the reduced Ca^2+^ transients in *ASAH1*^−/−^ iDA cells.

Loss of *ASAH1* results in increased levels of ceramide, as confirmed here by lipidomics in HeLa cells (Fig. 1a). Ceramides are known to affect the electron transport chain in mitochondria^9^, suggesting additional potential mechanisms independent of alterations in OXPHOS protein abundance. Interestingly, we also observed increased levels of BMP species in *ASAH1*^−/−^ HeLa cells. We speculate that increased levels of BMP may increase the efficiency of catabolism of sphingolipids in the context of *ASAH1* mutants, as indicated by reduced levels of GM2 and GM3 species. The tools developed here will allow a detailed analysis of lysosomal lipidomes and proteomes in iN and iDA cells lacking a broad set of LSD genes in future studies.

## METHODS

### Reagents

The following chemicals and reagents were used: 100×21mm Dish, Nunclon Delta (Thermo Fisher Scientific, 172931); 12 Well glass bottom plate with high performance #1.5 cover glass (Cellvis, P12-1.5H-N); 150 mm plates, 15 cm (MidSci, TP93150); 16% Paraformaldehyde, Electron-Microscopy Grade (Electron Microscopy Science, 15710); 2-Chloroacetamide (Sigma-Aldrich, C0267); 24 Well glass bottom plate with high performance #1.5 cover glass (Cellvis, P24-1.5H-N); 6 Well glass bottom plate with high performance #1.5 cover glass (Cellvis, P06-1.5H-N); 96 Well glass bottom plate with high performance #1.5 cover glass (Cellvis, P96-1.5H-N); Accutase (StemCell Technologies, 7920); Acetonitrile, Optima LC/MS Grade (Thermo Fisher Scientific, A955-4); Adenosine 5′ triphosphate, disodium, trihydrate (Thermo Fisher Scientific, 10326943); Ammonium formate CHROMASOLV LC-MS Ultra (Honeywell, 14266 Fluka); Anti-Flag M2 magnetic beads (Sigma Millipore, M8823; RRID: AB_2637089); B27 (Thermo Fisher Scientific, 17504001); Brain-derived neurotrophic factor, BDNF (PeproTech, 450-02); Caffeine (Tocris, 2793); CloneR (StemCell Technologies, 5889); CNQX (Cayman, 14618); cOmplete, EDTA-free Protease Inhibitor Cocktail (Millipore Sigma-Aldrich, 11873580001); Corning Matrigel Matrix (Corning, 354230); Corning 24 mm Transwell with 3.0 μm Pore Polycarbonate Membrane Insert, Sterile (Corning, 3414); Cover Glasses (VWR, 16004-308); Cultrex 3D Culture Matrix Laminin I (R&D Systems, 3446-005-01); D-AP5 (MedChemExpress, HY-100714A); DAPI (Thermo Fisher Scientific, D1306); Dextran, Alexa Fluor 647; 10 000 MW, Anionic, Fixable (Thermo Fisher Scientific, D22914); Dithiothreitol, DTT (Gold Biotechnology, DTT25); DMEM, High Glucose, Pyruvate (Thermo Fisher Scientific, 11995-073); DMEM/F12 (Thermo Fisher Scientific, 11330057); DNAse I (Thermo Fisher Scientific, EN0521); dNTPs (New England Biolabs, N0447L); Dounce homogenizer (DWK Life Sciences, 885302-0002); Doxycycline (Sigma-Aldrich, D9891); EPPS (Sigma-Aldrich, E9502); Fetal bovine serum (Cytiva, SH30910.03); FGF3 (in-house, N/A); Fluo-4, AM, cell permeant (Thermo Fisher Scientific, F14201); FluoroBrite DMEM (Thermo Fisher Scientific, A1896701); Formic Acid (Sigma-Aldrich, 94318); Geltrex LDEV-Free Reduced Growth Factor Basement Membrane Matrix (Thermo Fisher Scientific, A1413202); GlutaMAX (Thermo Fisher Scientific, 35050061); High-pH fractionation kit (Thermo Fisher Scientific, 84868); Hoechst33342 (Thermo Fisher Scientific, H1399); Holo-transferrin, human (Sigma-Aldrich, T0665); Human EGF Recombinant Protein (Cell Signaling Technology, 72528S); Human GDNF Recombinant Protein (PeproTech, 450-10-50UG); Human insulin (Sigma-Aldrich, I9278-5ML); Hydroxylamine solution (Sigma-Aldrich, 438227); Hygromycin B (Thermo Fisher Scientific, 10687010); Immobilon-P Membrane, PVDF, 0.45 μm (EMD Millipore, IPVH00010); Isopropanol, Optima LC/MS Grade (Thermo Fisher Scientific, A461-4); Lipofectamine LTX (Thermo Fisher Scientific, 15338100); Lys-C (Wako Chemicals, 129-02541); LysoTracker Red DND-99 (Thermo Fisher Scientific, L7528); MEM NEAA (Thermo Fisher Scientific, 11140050); ML-SA5 (MedChemExpress, HY-152182); N-2 Supplement (Thermo Fisher Scientific, 17502048); n-Butanol (Thermo Fisher Scientific, A383SK-4); NEAA (Life Technologies, 11140050); NEBNext Ultra II Q5 Master Mix (New England Biolabs, M0544L); Neurobasal (Thermo Fisher Scientific, 21103049); Neurotrophin-3, NT3 (PeproTech, 450-03); Nunc 12-well plate (Thermo Fisher Scientific, 150628); Nunc Cell-Culture Treated 6-well (Thermo Fisher Scientific, 140685); NuPAGE Novex 4-12% Bis-Tris Midi Protein Gels, 20 well (Thermo Fisher Scientific, WG1402BOX); NuPAGE Novex 4-12% Bis-Tris Midi Protein Gels, 26 well (Thermo Fisher Scientific, WG1403BOX); NuPAGE LDS Sample Buffer, 4X (Thermo Fisher Scientific, NP0008); Oligo dT20 primers (Invitrogen, 79654); OptiMEM I Reduced Serum Media (Thermo Fisher Scientific, 31985062); Oregon Green 488 BAPTA-1, AM, cell permeant (Thermo Fisher Scientific, O6807); Oregon Green 488 BAPTA-5N, Hexapotassium Salt, cell impermeant (Thermo Fisher Scientific, O6812); PBS (Corning, 21-031-CV); Penicillin-Streptomycin, 10 000 U/mL (Thermo Fisher Scientific, 15140163); PhosSTOP Phosphatase Inhibitor Cocktail (Roche, 4906845001); Pierce anti-HA magnetic beads (Thermo Fisher Scientific, 88837); Poly-L-ornithine hydrobromide (Sigma-Aldrich, P3655-50MG); Polyethylenimine (Polysciences, 23966); Precision Plus Protein Kaleidoscope Prestained Protein Standards (Bio-Rad, 1610395); Puromycin (Gold Biotechnology, P-600-500); Recombinant Human Sonic Hedgehog, Shh (PeproTech, 100-45); Recombinant SpCas9 (in-house, N/A); Revert 700 Total Protein Stain Kit (LI-COR, 926-11016); SDS (Bio-Rad, 1610302); Sep-Pak C18 cartridge (Waters, WAT054955); Sep-Pak tC18 96-well Plate, 25 mg Sorbent per Well (Waters, 186002319); SeraSil-Mag silica-coated superparamagnetic beads, 700 nm (Cytiva, 29357374); Silicon Dioxide film (Quantifoil, 50-192-7684); Silicon Dioxide R1/4 film (Quantifoil, 50-192-7684); SiR-Lysosome Kit (Cytoskeleton / Spirochrome, CY-SC012); SiR-Tubulin Kit (Cytoskeleton / Spirochrome, CY-SC002); Sodium bicarbonate (Sigma-Aldrich, S5761-500G); Sodium Pyruvate (Invitrogen, 11360070); Sodium selenite (Sigma-Aldrich, S5261-10G); STEMdiff Midbrain Neuron Differentiation Kit (StemCell Technologies, 100-0038); STEMdiff Midbrain Neuron Maturation Kit (StemCell Technologies, 100-0041); TCEP (Gold Biotechnology, TCEP2); Tetramethylrhodamine Ethyl Ester, Perchlorate, TMRE (Thermo Fisher Scientific, T669); TGF-beta (PeproTech, 100-21C); TMTpro 18plex Label Reagent (Thermo Fisher Scientific, A52045); Tris 1 M pH 8.0 RNase-free (Thermo Fisher Scientific, AM9855G); Tris(2-carboxyethyl)phosphine, TCEP (Gold Biotechnology, 51805-45-9); Trypsin (Promega, V511C); Trypsin-EDTA (Sigma-Aldrich, T4049-100ML); UltraPure 0.5 M EDTA pH 8.0 (Thermo Fisher Scientific, 15575020); Urea (Sigma-Aldrich, U5378); Uridine (Sigma-Aldrich, U3003); Vectashield (Vector Laboratories, H-1000-10); Water, Optima LC/MS Grade (Thermo Fisher Scientific, W64); Y-27632 Dihydrochloride ROCK inhibitor (PeproTech, 1293823); μ-Slide 8 Well Glass Bottom #1.5H Coverslip (ibidi, 80807); SpCas9^41^ and AsCas12a/AsCpf1^42^ were from a previous study ^21^.

The following antibodies were used: Anti-Actin (Sigma-Aldrich, A2228; RRID:AB_476697); Anti-Bassoon monoclonal antibody, SAP7F407 (Enzo, ADI-VAM-PS003-D); Anti-EEA1, C45B10 Rabbit mAb (Cell Signaling Technology, 3288S); Anti-EGF Receptor, D38B1 XP Rabbit mAb (Cell Signaling Technology, 4267); Anti-Flag antibody (Sigma-Aldrich, F1804); Anti-HA antibody (Cell Signaling Technology, 3724); Anti-HA High Affinity, rat IgG1 (Roche, 11867423001; RRID:AB_390918); Anti-LAMP1, D2D11 XP Rabbit (Cell Signaling Technology, 9091S; RRID:AB_2687579); Anti-LAMP1, D4O1S Mouse (Cell Signaling Technology, 15665; RRID:AB_2798750); Anti-Legumain (LGMN) antibody, recombinant (Abcam, ab183028); Anti-MAP2 Antibody (Cell Signaling Technology, 4542S); Anti-mouse IgG HRP conjugate (Bio-Rad, 170–6516; RRID:AB_11125547); Anti-Neurofilament heavy polypeptide antibody (Abcam, ab4680); Anti-Presenilin 2/AD5 antibody, PSEN2 (Abcam, ab51249); Anti-rabbit IgG HRP conjugate (Bio-Rad, 170–6515; RRID:AB_11125142); Anti-Synapsin I (SYN1) antibody [EPR23531–50], recombinant (Abcam, ab254349); Anti-Synaptophysin antibody [YE269] (Abcam, ab32127); Anti-Tubulin beta III antibody, chicken (Abcam, ab41489; RRID:AB_727049); Anti-Tyrosine Hydroxylase Antibody, clone LNC1 (Sigma-Aldrich, MAB318); Anti-VPS35 Antibody, B-5 (1:100, Santa Cruz Biotechnology, sc-374372); Donkey anti-Rat IgG (H+L) Highly Cross-Adsorbed Secondary Antibody, Alexa Fluor Plus 405 (Thermo Fisher Scientific, A48268; RRID:AB_2890549); Goat anti-Chicken IgY (H+L) Secondary Antibody, Alexa Fluor 488 (Thermo Fisher Scientific, A-11039; RRID:AB_2534096); Goat anti-Chicken IgY (H+L) Secondary Antibody, Alexa Fluor 647 (Thermo Fisher Scientific, A-21449; RRID:AB_2535866); Goat anti-Mouse IgG (H+L) Cross-Adsorbed Secondary Antibody, Alexa Fluor 488 (Thermo Fisher Scientific, A-11001; RRID:AB_2534069); Goat anti-Mouse IgG (H+L) Cross-Adsorbed Secondary Antibody, Alexa Fluor 647 (Thermo Fisher Scientific, A-21235; RRID:AB_2535804); Goat anti-Rabbit IgG (H+L) Cross-Adsorbed Secondary Antibody, Alexa Fluor 568 (Thermo Fisher Scientific, A-11011; RRID:AB_143157); Goat anti-Rabbit IgG (H+L) Cross-Adsorbed Secondary Antibody, Alexa Fluor 647 (Thermo Fisher Scientific, A27040; RRID:AB_2536101); Goat anti-Rabbit IgG (H+L) Highly Cross-Adsorbed Secondary Antibody, Alexa Fluor Plus 405 (Thermo Fisher Scientific, A48254; RRID:AB_2890548); Goat anti-Rabbit IgG (H+L) Highly Cross-Adsorbed Secondary Antibody, Alexa Fluor 488 (Thermo Fisher Scientific, A-11034; RRID:AB_2576217); Goat anti-Rat IgG (H+L) Cross-Adsorbed Secondary Antibody, Alexa Fluor 555 (Thermo Fisher Scientific, A-21434; RRID:AB_2535855); Goat anti-Rat IgG (H+L) Cross-Adsorbed Secondary Antibody, Alexa Fluor 647 (Thermo Fisher Scientific, A-21247; RRID:AB_141778); Transferrin from human serum, Alexa Fluor 488 conjugate (Thermo Fisher Scientific, T13342). All primary antibodies were used 1:500 for IFA and 1:1000 for WB (if not otherwise stated); secondary antibodies were used 1:200 for IFA and 1:5000 for WB.

### Cell culture of HeLa cell lines

HeLa TMEM192–3xHA cells (referred to as HeLa^TMEM192-HA^)^21^ were maintained in Dulbecco’s modified Eagle’s medium (DMEM), supplemented with 10% vol/vol fetal bovine serum (FBS), 5% vol/vol penicillin-streptomycin (P/S), 5% vol/vol GlutaMAX and 5% vol/vol non-essential amino acids (NEAA) at 37°C, 5% O_2_. Unless otherwise noted, we refer to independently grown and handled cultures as biological replicates to distinguish from assays performed on identical samples (i.e. technical replicates).

### Stem-cell culture and neuronal differentiation

Detailed methods for maintenance and differentiation of stem cells can be found at: dx.doi.org/10.17504/protocols.io.br85m9y6, dx.doi.org/10.17504/protocols.io.br9cm92w, dx.doi.org/10.17504/protocols.io.br9em93e, dx.doi.org/10.17504/protocols.io.bsacnaaw. Briefly, human ES cells (H9, WiCell Institute) were cultured in E8 medium^43,44^ on Geltrex-coated tissue culture plates with daily medium change. Cells were passaged every 4–5 days with 0.5 mM EDTA in 1× DPBS (Thermo Fisher Scientific).

For conversion of human stem cells to iNeurons, cells were expanded and plated at 2×10^4^/cm^2^ on Geltrex-coated tissue plates in DMEM/F12 supplemented with 1x N2, 1x NEAA (Thermo Fisher Scientific), human Brain-derived neurotrophic factor (BDNF, 10 ng/ml, PeproTech), human Neurotrophin-3 (NT-3, 10 ng/ml, PeproTech), mouse laminin (0.2 μg/ml, Cultrex), Y-27632 (10 μM, PeproTech) and Doxycycline (2 μg/ml, Alfa Aesar) on Day 0. On Day 1, Y-27632 was withdrawn. On Day 2, medium was replaced with Neurobasal medium supplemented with 1x B27 and 1x Glutamax (Thermo Fisher Scientific) containing BDNF, NT-3 and 1 μg/ml Doxycycline. Starting on Day 4, half of the medium was replaced every other day thereafter. On Day 7, the cells were treated with 0.5μM EDTA + PBS and plated on Geltrex-coated tissue plates. Following day 12 of differentiation 50% ND2 media was changed every other day. Cells were replated at day 16 into triple-coated dishes (Geltrex + poly-L-orthinine + laminin). After day 26, 50% of media was changed every 4 days.

For human ES cell conversion to induced dopaminergic neurons (iDA neurons), were differentiated as described^45^. Cells were seeded on Geltrex-coated plates at DIV-2 and expanded in standard culture medium. On Day 0 (DIV 0), medium was replaced with DMEM/F12-based D0/1 induction medium supplemented containing N2, B27, NEAA, BDNF, GDNF, mouse laminin, and doxycycline (2 μg/ml) to induce NGN2 expression. On Day 1, the D0/1 induction medium (+dox) was refreshed. On Day 2 (DIV 2), cells were washed with PBS and switched to iDA differentiation medium supplemented with 2 μM Ara-C to inhibit proliferating cells. Media was changed daily from DIV 2 to DIV 4. From DIV 5 to DIV 8, half of the medium was replaced daily with fresh iDA differentiation medium. On Day 9, cells were transitioned to midbrain maturation medium containing doxycycline. From DIV 10 onward, half of the maturation medium was replaced every other day until terminal differentiation (e.g., DIV 26). Cells were replated at day 16 into triple-coated dishes (Geltrex + poly-L-ornithine+ laminin). After day 26, 50% of media was changed every 4 days.

### Cell culture for neuronal soma and projection fractions

To generate samples for soma and neuronal projection proteome, H9-NGN2 stem cells were differentiated as described above but seeded on day 4 onto Transwell insets (Corning, two insets per replicate, 3 μm pore) ^46^. Membranes were coated on both sides with Geltrex before using for the experiment. Media was changed in both compartments. Media changes were performed on both the bottom and top part of the 6 well inset. On the day 35, media was aspirated and the projections at the bottom of the membrane (== neuronal projections) cut off with a clean razor and collected in sterile 1xPBS + protease inhibitor cocktail (Roche), before colleting the top fraction on the membrane (== soma). Samples were pelleted at 4°C for 5 min at 5000g, supernatant aspirated and resuspended in 8M Urea lysis buffer (+ protease inhibitor cocktail) for subsequent standard sample preparation for TMTpro (see below).

### Gene-Editing

A protocol for gene editing of LSDs can be found at: dx.doi.org/10.17504/protocols.io.dm6gpmdwpgzp/v1. Generation of LSD mutants in H9^NGN2;TMEM192-HA^ cells^21,25^ was facilitated using CRISPR/Cas9 with target sites determined using CHOPCHOP (https://chopchop.cbu.uib.no/). Briefly, 0.6 μg sgRNA (Supplementary Table 3) was incubated with 3 μg SpCas9 or Cpf1 protein for 10 minutes at room temperature and electroporated into 2×10^5^ H9^NGN2;TMEM192-HA^ cells using Neon transfection system (Thermo Fisher Scientific) and sorted into 96-well dishes containing 300 μL full growth medium (composition as described above). Single cells were allowed to grow into colonies and duplicated for multiplex sequencing. Genomic DNA samples were obtained by incubating cells in 30 μL PBND (50 mM KCl, 10 mM Tris-HCl, pH 8.3, 2.5 mM MgCl_2_-6H_2_O, 0.45% NP-40 and 0.45% Tween-20) with protease K (40 μg/ml) at 37°C for 5 min and heated to 55°C and 95°C for 30min and 15 min, respectively. The first round of PCR was performed to amplify the target region using gene-specific primers (Supplementary Table 3) that contain partial Illumina adaptor sequences (i.e., Forward primer: 5’-ACACTCTTTCCCTACACGACGCTCTTCCGATCT[n]_18–22_ -3’, Reverse primer: 5’- GTGACTGGAGTTCAGACGTGTGCTCTTCCGATCT[n]_18–22_ -3’, [n]_18–22_ represent gene specific sequences). The resulting PCR products with adapter-modified ends can be further amplified in the second round of PCR by universal primers containing attachment sites for the flow cell and index sequences (i.e., Forward primer: 5’- AATGATACGGCGACCACCGAGATCTACACTCTTTCCCTACACGACGCTCTTCCGATCT -3’, Reverse primer: 5’- CAAGCAGAAGACGGCATACGAGAT-[n]_8_-GTGACTGGAGTTCAG ACGTGTGCT -3’, [n]_8_ represents index sequences). The final PCR products were purified using QIAquick PCR purification kit (Qiagen, 28106). Sequencing was performed using Miseq Reagent kits v2 on Illumina Miseq following the denature and dilute libraries guide of Miseq system, and out-of-frame indels were identified using OutKnocker (www.OutKnocker.org)^47^. Knockout candidates were confirmed by Western blot on whole cell lysates or by proteomics (Supplementary Table 3) and individual deletion sequences are provided in Supplementary Table 3. HeLa^TMEM192-HA^ Control as well as Control, *NPC1*^−/−^ and *NPC2*^−/−^ H9^NGN2;TMEM192-HA^ cells have been previously reported^21^.

### Organelle IP from HeLa Cells

For LysoIP from HeLa cells, we employed a previously reported method: dx.doi.org/10.17504/protocols.io.bw7hphj6. Briefly, HeLa cells (untagged, Control, and *ASAH1*^−/−^) were seeded in three 10 cm dishes per replicate and harvested at 70–80 % confluency. For LysoIP, 65 μL of anti-HA magnetic bead slurry (Pierce) was used per replicate, and samples were incubated with beads at 4 °C with gentle rotation for 30 min. From each incubation, 10 μL of flow-through was collected for Western blot analysis. Anti-HA beads were washed twice with high-salt KPBSi (150 mM NaCl) and once with KPBSi. Samples were eluted with 120 μL 0.5 % NP-40 in KPBSi at 4°C with gentle rotation for 30 min. From each eluate, 20 μL was used for Western blot analysis, and 100 μL was further processed for TMTpro proteomics (see below).

### Lipidomics

For lipidomics of whole-cell and isolated lysosomes from HeLa cells, 5×15cm dishes per genotype (untagged, Control and *ASAH1*^−/−^) were used as input for LysoIP and 6 wells of a 6 well per genotype for whole-cell samples. Whole-cell samples were washed three times with 1xPBS, and harvested in ice-cold 1xPBS (+ protease inhibitor cocktail) by cell scraping. Cells were pelleted, the supernatant aspirated and snap frozen. LysoIP was performed as described above with the modification that after the IP, HA-beads were washed, snap frozen and shipped for lipidomic analysis.

For lipid extraction, whole cell or LysoIP samples were processed in ice-cold milli-Q water by snap-freezing (in liquid nitrogen)/thawing (using an ultrasound water bath for 3 min) repeatedly and finally extracted as described^48^. Protein concentration in the lysates was quantified using the BCA assay, and approximately 50 μg of lysate was transferred to Pyrex glass tubes with PTFE-lined caps. For lipid extraction, 6 mL of ice-cold chloroform and methanol (2:1 v/v) and 1.5 mL of water were added to each sample. The tubes were thoroughly vortexed to ensure homogeneous mixing of polar and non-polar solvents. SPLAH internal standards (Avanti Product Number 330707–1EA) were added before extraction. The samples were centrifuged at 1000 rpm for 20 minutes at 4°C to separate the organic and aqueous phases. The lower organic phase was carefully pipetted into a new glass tube using a sterile glass pipette, avoiding the intermediate layer containing cell debris and precipitated proteins. The organic phase was then dried under a nitrogen stream until all solvents had evaporated. Finally, the samples were reconstituted in 100 μL of Isopropanol: Acetonitrile: Water (60:35:5) and stored at −80°C prior to analysis.

For ganglioside extraction, the aqueous layer was collected and dried under a gentle stream of nitrogen. The dried aqueous phase was reconstituted in 1 mL pure water and subsequently desalted by using Sola HRP SPE 30 mg/2 mL 96-well plate 1EA (Thermo Scientific #60509–001). Initially, the cartridges were cleaned 3 times with 1 mL of MeOH and equilibrated 3 times with water. Samples were loaded onto the column, washed 3 times with water and, finally, the gangliosides were eluted by three times 1 mL of MeOH. The eluate was dried under nitrogen flow and reconstituted in MeOH/H2O/CHCl3 (60:9:120, v/v/v).

For LC-MS/MS analysis, lipids were separated using ultra-high-performance liquid chromatography (UHPLC) coupled with tandem mass spectrometry (MS/MS). UHPLC analysis was conducted on a C30 reverse-phase column (Thermo Acclaim C30, 2.1 × 250 mm, 3 μm) maintained at 55°C and connected to a Vanquish Horizon UHPLC system, along with an OE240 Exactive Orbitrap MS (Thermo Fisher Scientific) equipped with a heated electrospray ionization probe. Each sample (5 μL) was analyzed in both positive and negative ionization modes. The mobile phase included 60:40 water: acetonitrile with 10 mM ammonium formate and 0.1% formic acid, while mobile phase B consisted of 90:10 isopropanol: acetonitrile with the same additives. The elution was performed with a gradient of 90 min; for 0–7 min, elution started with 40% B and increased to 55%; from 7 to 8 min, increased to 65% B; from 8 to 12 min, elution was maintained with 65% B; from 12 to 30 min, increased to 70% B; from 30 to 31 min, increased to 88% B; from 31 to 51 min, increased to 95% B; from 51 to 53 min, increased to 100% B; during 53 to 73 min, 100% B was maintained; from 73 to 73.1 min, solvent B was decreased to 40% and maintained for another 16.9 min for column re-equilibration. Flow rate of 0.2 mL/min, injection volume of 5 μL, and column temperature of 55°C. Mass spectrometer settings included an ion transfer tube temperature of 300°C, vaporizer temperature of 275°C, Orbitrap resolution of 120,000 for MS1 and 30,000 for MS2, RF lens at 70%, with a maximum injection time of 50 ms for MS1 and 54 ms for MS2. Positive and negative ion voltages were set at 3250 V and 2500 V, respectively. Gas flow rates included auxiliary gas at 10 units, sheath gas at 40 units, and sweep gas at 1 unit. High-energy collision dissociation (HCD) fragmentation was stepped at 15%, 25%, and 35%, and data-dependent tandem MS (ddMS2) ran with a cycle time of 1.5 s, an isolation window of 1 m/z, an intensity threshold of 1.0 × 10^4^, and a dynamic exclusion time of 2.5 s.

Full-scan mode with ddMS^2^ was performed over an m/z range of 250–1700, with EASYIC^™^ (ThermoFischer) used for internal calibration. The raw data were processed and aligned with LipidSearch 5.1 (ThermoFischer, #OPTON 30879), using a precursor tolerance of 5 ppm and a product tolerance of 8 ppm. Further filtering and normalization were conducted using Lipidcruncher^49^. Semi-targeted quantification was performed by normalizing the area under the curve (AUC) to the AUC of internal standards and further normalized with the total quantified protein level.

For LC-MS/MS analysis of gangliosides, samples were analyzed using a Vanquish UHPLC system (Thermo Scientific) coupled to an Orbitrap Exploris 240 mass spectrometer (Thermo Scientific, #BRE725535). Separation was achieved on a Kinetex HILIC column (Phenomenex, #00D-4461-AN; 2.6 μm, 100 × 2.1 mm). The mobile phase consisted of solvent A (acetonitrile with 0.2% v/v acetic acid) and solvent B (water containing 10 mM ammonium acetate, pH 6.1, adjusted with acetic acid). The column temperature was maintained at 50 °C. A gradient elution was employed at a constant flow rate of 0.6 mL / min: 12.3 % B at 0 min, a linear increase to 22.1% B from 1 to 15 min, followed by column equilibration at 12.3% B for 5 minutes. Mass spectrometry analysis was performed in heated electrospray ionization (HESI) mode under the following conditions: spray voltage at −4.5 kV, heated capillary temperature at 300°C, and vaporizer temperature at 250°C. The gas settings were as follows: sheath gas at 40 units, auxiliary gas at 5 units, and sweep gas at 1 unit. The ion transfer tube temperature was maintained at 300°C. For MS1 analysis, the Orbitrap resolution was set to 120,000 with a scan range of 700–1800 m/z, RF lens at 60%, and an AGC target set to standard. For MS2, the Orbitrap resolution was set to 30,000. Internal calibration was achieved using EASY-IC. After data search as above, quantification was achieved by normalizing the area under the curve to GM3-d5 standards, followed by further normalization to the amount of protein used in the preparation.

### Label-free nDIA proteomics of iNeurons and iDA neurons

Control and LSD mutant cells were seeded on Geltrex-coated tissue culture plates and differentiated according to the methods stated above. For d30 timepoints, Control, *GRN*^−/−^, *GBA1*^−/−^, *ASAH1*^−/−^, *SMPD1*^−/−^ cells were seeded in triplicates in coated 12 well plates. For all other timepoints, cells were replated at day 16 in triplicates into coated 12 well plates. Cells were dissociated using 0.5 mM EDTA-PBS and washed in PBS, pelleted at 2000g for 5 min, the supernatant aspirated and pellet snap frozen and stored at −80°C.

Cell pellets were lysed in 140 μL 8M Urea and 100 mM Tris pH 8.0 by syringe with a 21G needle. Protein concentration was measured by protein BCA assay. 10 mM TCEP and 40 mM chloroacetamide were added for reduction and alkylation. LysC (FUJIFILM Wako) was added to samples in a 100:1 ratio (protein-to-LysC) and the samples were incubated on a rocker for 4 h at room temperature. The urea was diluted to 2 M with 100 mM Tris pH 8.0. Trypsin was added to samples in a 100:1 ratio (protein-to-trypsin) and the samples were incubated on a rocker overnight at room temperature. Digested peptides were desalted using Waters 25 mg Sep-Pak tC18 96-well Plates (Cat. No.: 186002319). The concentration of desalted peptides was measured by peptide BCA assay.

Separation was performed on a C18 capillary column (40 cm length × 100 μm inner diameter) packed with Accucore resin (2.6 μm, 150 Å, Thermo Fisher Scientific) at 55 °C and an Vanquish Neo LC system (Thermo Scientific). Flow rate was set to 450 nL/min. Mobile phase A consisted of 0.125% formic acid in ACN/water (5:95, v/v). Mobile phase B consisted of 0.125% formic acid in ACN/water (95:5, v/v). When peptides were loaded on the column, mobile phase B increased from 5% to 35% over 32 minutes, increased to 99% over 2 minutes, and stayed at 99% for 6 minutes. Eluting peptides were analyzed by an Orbitrap Astral mass spectrometer (Thermo Scientific) using nDIA^39,50^ and analyzed by DIA-NN (v2.1.0)^51,52,53^. Methionine oxidation and N-terminal acetylation were enabled as variable modifications. Precursor m/z range was set to 380 to 980. Fragment ion m/z range was set to 110 to 2000. All other settings were kept default.

### TMTpro 18plex proteomics

#### Proteomic sample preparation.

Sample preparation of proteomic analysis of whole-cell extract from HeLa control and mutant lysates performed according to previously published studies^21,44^. Replicate cell cultures were grown and treated independently and are considered biological replicates in the context of TMT experiments. Cells were washed twice with 1xPBS and harvested on ice using a cell scraper in 1xPBS. Cells were pelleted via centrifugation for 5 minutes (5000*g*, 4°C), and washed with 1xPBS before resuspending in lysis buffer (Urea, 150 mM TRIS pH 7.4, 150mM NaCl, protease and phosphatase inhibitors added). After a 10 second sonication, and optional French-pressing through a G25 needle, lysed cells were pelleted and protein concentration of clarified sample determined using BCA kit (Thermo Fisher Scientific, 23227). 100 μg protein extract of each sample were incubated for 30 minutes @ 37°C with 5 mM TCEP for disulfide bond reduction with subsequent alkylation with 25 mM chloroacetamide for 10 minutes at RT with gentle shaking. Methanol-chloroform precipitation of samples was performed as follows: To each sample, 4 parts MeOH was added, vortexed, one part chloroform added, vortexed, and finally 3 parts water added. After vortexing, suspension was centrifugated for 2 minutes at 14000*g* and the aqueous phase around the protein precipitate removed using a loading tip. Peptides were washed twice with MeOH and resuspended in 200 mM EPPS, pH 8, and digested for 2 h with LysC (1:100) at 37°C, followed by Trypsin digestion (1:100) at 37°C overnight with gentle shaking.

#### Tandem mass tag (TMT) labeling.

50 μL of digested samples were labeled by adding 10 μL of TMT reagent (stock: 20 mg/ml in acetonitrile, ACN) together with 10 μL acetonitrile (final acetonitrile concentration of approximately 30% (v/v)) for 2 h at room temperature before quenching the reaction with hydroxylamine to a final concentration of 0.5% (v/v) for 15 minutes. The TMTpro-labeled samples were pooled together at a 1:1 ratio, resulting in consistent peptide amount across all channels. Pooled samples were vacuum centrifuged for 1 hour at room temperature to remove ACN, followed by reconstitution in 1% FA, samples were desalted using C18 solid-phase extraction (SPE) (200 mg, Sep-Pak, Waters) and vacuum centrifuged until near dryness.

#### Basic pH reverse phase HPLC.

Dried peptides were resuspended in 10 mM NH_4_HCO_3_ pH 8.0 and fractionated using basic pH reverse phase HPLC^54^. Samples were offline fractionated into 96 fractions over a 90 minutes run by using an Agilent LC1260 with an Agilent 300 Extend C18 column (3.5 μm particles, 2.1 mm ID, and 250 mm in length) with mobile phase A containing 5% acetonitrile and 10 mM NH_4_HCO_3_in LC-MS grade H_2_O, and mobile phase B containing 90% acetonitrile and 10 mM NH_4_HCO_3_ in LC-MS grade H_2_O (both pH 8.0). The 96 resulting fractions were then pooled in a non-continuous manner into 24 fractions^55^. This set of 24 fraction was divided into 2×12 sets (even or odd numbers), acidified by addition of 1% Formic Acid (FA) and vacuum centrifuged until near dryness. One set (12 samples) was desalted via StageTip, dried and reconstituted in 10 μL 5% ACN, 5% FA before LC-MS/MS processing.

#### Mass spectrometry acquisition.

For TMTpro proteomics, data collection was performed on a Orbitrap Eclipse Lumos Tribrid mass spectrometer (for whole-cell iN and iDA) or Orbitrap Ascend Tribid mass spectrometer (for HeLa LysoIP and neuronal whole-cell, soma and projection proteome) (Thermo Fisher Scientific, San Jose, CA), coupled with a FAIMS Pro device and a Proxeon EASY-nLC1200 liquid chromatography (Thermo Scientific). 10% of resuspended samples were loaded on a 35 cm analytical column (100 mm inner diameter) packed with Accurcore150 resin (150 Å, 2.6 mm, Thermo Fisher Scientific, San Jose, CA) for LC-MS analysis. Peptide separation was performed with a gradient of acetonitrile (ACN, 0.1% FA) from 3–13% (0–83 minutes) and 13–28% (83–90 minutes) during a 90 min run. LC-MS/MS was combined with 3 optimized compensation voltages (CV) parameters on the FAIMS Pro Interface to reduce precursor ion interference^56^. Data-dependent acquisition (DDA) was performed by selecting the most abundant precursors from each CV’s (−40/−60/−80) MS1 scans for MS/MS over a 1.25 second duty cycle. The parameters for MS1 scans in the Orbitrap include a 400–1,600 m/z mass range at 60,000 resolution (at 200 Th) with 4 × 10^5^ automatic gain control (AGC) (100%), and a maximum injection time (max IT) of 50 ms. Most abundant precursors (with 120 s dynamic exclusion +/− 10 ppm) were selected from MS1 scans, isolated using the quadrupole (0.6 Th isolation), fragmented with higher-energy collisional dissociation (HCD, 36% normalized collision energy), and subjected to MS/MS (MS2) in the Orbitrap detector at 50,000 resolution, 5x AGC, 110 – 200 m/z mass range, IT 86 ms and with 120 s dynamic exclusion +/− 10 ppm.

#### Data processing.

Raw mass spectra were converted to mzXML, monoisotopic peaks reassigned using Monocle^57^ and searched using Comet^58^ against all canonical isoforms found in the Human reference proteome database (UniProt Swiss-Prot 2019–01; https://ftp.uniprot.org/pub/databases/uniprot/previous_major_releases/release-2019_01/)) as well as against sequences from commonly found contaminant proteins and reverse sequences of proteins as decoys, for target-decoy competition ^59^. For searches, a 50-ppm precursor ion tolerance and 0.02 Da product ion tolerance for ion trap MS/MS as well as trypsin endopeptidase specificity on C-terminal with 2 max. missed cleavages was set. Static modifications were set for carbamidomethylation of cysteine residues (+57.021 Da) and TMTpro labels on lysine residues and N-termini of peptides (+304.207 Da); variable modification was set for oxidization of methionine residues (+15.995 Da). Peptide-spectrum matches (PSMs) were filtered at 2% false discovery rate (FDR) using linear discriminant analysis (Picked FDR method, based on XCorr, DeltaCn, missed cleavages, peptide length, precursor mass accuracy, fraction of matched product ions, charge state, and number of modifications per peptide (additionally restricting PSM Xcorr >1 and peptide length>6, and after a 2% protein FDR target filtering^60^ PSM reporter ion intensities were quantified. Quantification was performed using a 0.003-Da window around the theoretical TMT-reporter m/z, and filtered on precursor isolation specificity of > 0.5 in the MS1 isolation window and the output was filtered using summed SNR across all TMT channels > 200. MSstatsTMT^61^ was performed on peptides with >200 summed SNR across TMT channels. For each protein, the filtered peptide–spectrum match TMTpro raw intensities were summed and log_2_ normalized to create protein quantification values (weighted average) and normalized to total TMT channel intensity across all quantified PSMs (adjusted to median total TMT intensity for the TMT channels)^62^. Log_2_ normalized summed protein reporter intensities were compared using a Student’s t-test and p-values were corrected for multiple hypotheses using the Benjamini-Hochberg adjustment^63^. Subcellular and functional annotations were based on previous published list of high confidence annotations ^64^, “high” & “very high” confidence, additional manual entries from^44,65^, AmiGO Pathway online tool and mitochondrial annotation was based on MitoCarta 3.0^66^. GO-enrichment was performed with ShinyGO^67^ or topGO.

### Analysis of proteomic datasets

Resulting files were analysed using R scripts, which is available on GitHub (https://github.com/sauerkrausi/neuroLSD).

#### HeLa LysoIP TMT Proteomics:

We analyzed LysoIP-TMT data from HeLa cells (untagged, HeLa^TMEM192-HA^ control, *ASAH1*^−/−^; in triplicates) quantified with MSstats. Results were transformed to −log10(p/q), filtered for finite log2FCs, and joined to a curated subcellular annotation matrix with hydrolase genes flagged. Row-z heatmaps were generated by genotype and fraction, and cluster-level patterns were visualized with violin plots. Organelle-specific co-enrichment was tested relative to lysosomal means, followed by Wilcoxon tests with Benjamini–Hochberg correction. Overlap structure was visualized by Venn diagrams, and enrichment of biological processes was assessed with topGO.

#### LFQ-nDIA of iN and iDA neurons:

Whole-cell proteomes from iN and iDA neurons (day 30 and 50; in triplicates per neuron and genotype) were analyzed for quality control, PCA, heatmaps, and circos visualizations. Median RSD and protein counts were computed per neuron type and genotype. Fold-change matrices were clustered, joined with subcellular annotations, and used for Euler diagram overlap analyses. Knockout efficiency for key lysosomal genes was verified by barplots and heatmap

#### TMT Proteomics of Day 23 Neurons:

MSstats output from iN and iDA neurons (Ctrl, *ASAH1*^−/−^, *SMPD1*^−/−^; in triplicates) was transformed to −log10(p/q) and merged with binary subcellular annotations. Visualization included heatmaps, KO×KO correlation matrices, scatterplots, volcano plots, and violin plots of cluster-level log_2_FC distributions. GO enrichment was performed per cluster using topGO, with dot plots summarizing significant terms.

#### TMT Proteome of whole-cell, soma and neuronal projections (Day 35):

TMT-18plex proteomes from whole-cell, soma, and neuronal projections fractions (Ctrl and *ASAH1*^−/−^; in triplicates) were processed as above. We computed per-annotation mean log_2_FCs, internally normalized axon-versus-whole-cell contrasts, and a ratio-of-ratios (RoR) metric to classify significant shifts. Annotation effects were ranked by delta mean, Cohen’s d, and RoR magnitude. Top-ranked sets were tested by Wilcoxon, visualized with Euler overlaps, and analyzed for GO enrichment.

### Light Microscopy

#### Live-cell spinning disk microscopy – general acquisition parameters.

For live-cell spinning disk microscopy of organelles, cells were seeded into either 24-well 1.5 high-performance glass-bottom plates (Cellvis, P24–1.5H-N) or μ-Slide 8-well glass-bottom plates (ibidi, #80807) and cultured in the same vessels until reaching the appropriate confluency. Prior to imaging, cells were washed with 1× PBS and imaged in FluoroBrite DMEM medium. Imaging was performed on a Yokogawa CSU-X1 spinning disk confocal mounted on a Nikon Eclipse Ti2-E motorized microscope equipped with a Tokai Hit stage-top incubator. Conditions were maintained at 37 °C, 5% CO_2_, and 95% humidity using a Nikon Plan Apo 60×/1.40 N.A. immersion oil objective lens. Fluorophores were sequentially excited with a Nikon LUN-F XL solid-state laser combiner using the following settings ([laser line – laser power]): 405 nm – 80 mW, 488 nm – 80 mW, 561 nm – 65 mW, and 640 nm – 60 mW, with a Semrock Di01-T405/488/568/647 dichroic mirror. Emissions were collected through Chroma ET455/50m [405 nm], Chroma ET525/36m [488 nm], Chroma ET605/52m [561 nm], and Chroma ET700/75m [640 nm] filters (Chroma Technologies). Images were acquired using a Hamamatsu ORCA-Fusion BT CMOS camera (6.5 μm^2^ photodiode, 16-bit) and NIS-Elements software. Identical laser intensities and exposure times were applied across all samples. Brightness and contrast were adjusted equally by applying the same minimum and maximum display values in ImageJ/Fiji. Image quantification was performed in ImageJ/Fiji using custom-written batch macros.

#### Ca^2+^-imaging in iNeurons and iDA neurons using spinning disk microscopy.

iNeurons and iDA neurons were differentiated to the indicated time points in μ-Slide 8-well glass-bottom plates (ibidi, #80807). On the day of measurement, cells were incubated with Fluo-4 for 30 min at 37 °C, washed with warm PBS, and fresh ND2 medium was added. Neurons were imaged at 37 °C and 5% CO_2_ using a Nikon Eclipse Ti2-E motorized spinning disk confocal microscope (as described above) with a Nikon Plan Lambda 20×/0.75 N.A. air objective in 2×2-pixel binning mode (785×570 px). For each time-lapse, 1000 frames were recorded at 10 frames/s (100 ms exposure at 5% 488 nm laser power). Calcium dynamics were recorded under basal conditions, stimulation with 200 mM KCl, or inhibition with 50 μM CNQX and 20 μM D-AP5.

Images were analyzed using cellposeSAM and R with custom-trained models and scripts. Time-lapses were segmented with custom ML models in cellposeSAM, and the resulting masks were matched to fluorescence intensity over time for each ROI. Traces were baseline-corrected (ΔF/F), binarized, and evaluated for network synchrony and event dynamics. Batch processing enabled per-cell trace extraction and correlation analysis.

#### Immunocytochemical analysis.

HeLa cells were fixed with warm 4 % paraformaldehyde (Electron Microscopy Science, #15710, purified, EM grade) in PBS at 37°C for 30 min and permeabilized with 0.5 % Triton X-100 in PBS for 15 min at room temperature. After three washes with 0.02% Tween20 in PBS (PBST), cells were blocked for 10 minutes in 3 % BSA-1xPBS at room temperature and washed again three times in PBST. Cells were incubated for 3 h in primary antibodies in 3 % BSA-1xPBS and washed three times with PBST. Secondary antibodies (Thermo Scientific, 1:400 in 3 % BSA-1xPBS) where applied for 1 hour at room temperature. To stain nuclei, Hoechst33342 (1:10000) was added for 5 minutes to cells in PBST and finally washed three times.

#### Fixed-cell microscopy – general acquisition parameters.

Immunofluorescently labelled Hela or iNeurons were imaged at room temperature using a Yokogawa CSU-W1 spinning disk confocal on a Nikon Eclipse Ti2-E motorized microscope equipped with a Nikon Plan Apochromat 40×/0.40 N.A air-objective lens, Nikon Plan Apochromat 60×/1.42 N.A oil-objective lens and a Plan Apochromat 100×/1.45 N.A oil-objective lens. Signals of 405/488/568/647 fluorophores were excited in sequential manner with a Nikon LUN-F XL solid state laser combiner ([laser line – laser power]: 405 nm - 80 mW, 488 nm - 80 mW, 561nm - 65 mW, 640 nm - 60 mW using a Semrock Di01-T405/488/568/647 dichroic mirror. Fluorescence emissions were collected with Chroma ET455/50m [405 nm], 488 Chroma ET525/50m [488 nm], 568 Chroma ET605/52m [561 nm], 633 Chroma ET705/72m [640 nm] filters, respectively (Chroma Technologies). Confocal images were acquired with a Hamamatsu ORCA-Fusion BT CMOS camera (6.5 μm^2^ photodiode, 16-bit) camera and NIS-Elements image acquisition software. Consistent laser intensity and exposure time were applied to all the samples, and brightness and contrast were adjusted equally by applying the same minimum and maximum display values in ImageJ/FiJi^68^.

#### Evaluation of endo-lysosomal morphology in HeLa cell lines.

For the quantitative measurement of select endo-lysosomal markers (EEA1, VPS35, RAB7, LAMP1, TMEM192), maximum-intensity projection images were analyzed with a custom Python pipeline. DNA nuclei were segmented with CellposeSAM to enable per-nucleus normalization. Organelle channels were preprocessed by rank-mean background subtraction and white-tophat enhancement, with additional wavelet denoising for VPS35, followed by Otsu thresholding and area filtering based on equivalent-diameter bounds. For each field, object counts, occupied area, and average object area were computed per marker and normalized to nuclei counts. Pairwise colocalization among non-DNA markers was assessed by centroid proximity within a fixed submicron radius, and results were saved as QC overlays and CSV summaries per image, including a table of pairwise colocalization metrics for downstream analysis.

#### TFN-uptake and colocalization.

The quantitative measurement of transferrin uptake (TFN-488) and its colocalization with early endosomes (EEA1) in HeLa cells was performed using a custom Python pipeline. Maximum-intensity projection channel pairs were identified per replicate by filename parsing, converted to 8-bit, background-corrected with rank-mean filtering (disk 10–15 px), and sharpened by white-tophat (disk 3). Marker-positive objects were segmented by Otsu thresholding with area filtering (30–2000 px). For each field, object counts and occupied area for EEA1 and TFN were computed and normalized to image area. Pairwise colocalization was assessed by centroid proximity within a fixed submicron radius (~3.1 px), and results were saved as QC overlays and CSV summaries per image and replicate for downstream analysis.

#### Evaluation of TH positive neurons.

Time-lapse calcium imaging data in ND2 format were processed using a custom Python pipeline implementing CellposeSAM segmentation. Image stacks were read with the nd2 package, and maximum intensity projections were generated prior to segmentation with a pretrained Cellpose model optimized for neuronal soma. Segmentation masks were filtered by area (100–10,000 pixels) to exclude artifacts and outliers. For each valid region of interest, centroid coordinates were extracted, and mean fluorescence intensities were computed across all time frames. Segmentation results were saved as 16-bit TIFF masks and visualization overlays, while per-cell centroid coordinates and intensity traces were exported as CSV files for downstream analysis and plotting in R.

#### Evaluation of synaptic proteins in iN and iDA samples.

The quantitative measurement of select synaptic markers (for example, SYP, Bassoon) in iN and iDA neurons was performed using a custom Python pipeline. Maximum-intensity projection images were parsed for experimental metadata, and cytoskeletal channels were used to generate neuronal masks. Marker-specific background subtraction and segmentation routines were applied to detect synaptic puncta, followed by measurement of object morphology and fluorescence intensity. Colocalization between SYP and Bassoon was evaluated at the object level, and results were exported as cleaned images, QC overlays, and CSV files containing per-object and per-image metrics for downstream analysis.

### Electron Microscopy

iNeuron and iDA cells of Control and select mutant cells were grown on Aclar plastic coverslips in above stated growth conditions until 70–80 % confluency was reached, washed twice in 1x PBS and fixed with a fixation mixture of 2 % formaldehyde and 2.5 % glutaraldehyde in 0.1 M Sodium Cacodylate buffer, pH 7.4 for 1 hour at room temperature. Sample preparation and microscopy was performed by the Harvard Medical School Electron microscopy facility (https://electron-microscopy.hms.harvard.edu/methods).

### Cryo-ET

Associated protocols can be found on protocols.io (https://www.protocols.io/view/sample-preparation-and-vitrification-of-cell-cultu-yxmvme659g3p/v1, https://www.protocols.io/view/cryo-plasma-focused-ion-beam-pfib-milling-e6nvw1rozlmk/v1, https://www.protocols.io/view/cryo-et-data-acquisition-tomogram-reconstruction-a-dm6gpznxdlzp/v1).

#### Sample preparation and freezing.

HeLa^TMEM192-HA^ Control and *ASAH1*^−/−^ cells were cultured on EM grids as follows: 200-mesh gold grids with Silicon Dioxide R1/4 film (Quantifoil) were plasma cleaned, coated by incubation with 0.25 μg/mL laminin (Sigma-Aldrich, L4544) for 1h under the UV light of a sterile environment and afterwards washed twice with PBS. One day before plunging, laminin-coated grids are transferred to 8-well μ-Slide dishes (Ibidi, 80826) and ~12,000 cells are seeded per well together with 0.1mg/mL 10kDa Dextran coupled to Alexa Fluor 647 (Thermo Fisher Scientific, D22914) for endolysosome detection under cryogenic conditions. The next day, grids were vitrified with 3.5 μL of PBS-diluted autofluorescent 1 μm diameter Dynabeads (Thermo Fisher Scientific, acid65011) after 6.5s blotting time at 37°C and 70% relative humidity in liquid ethane (at −184°C) using an EM GP2 plunger (Leica Microsystems). After plunging, the grids were clipped into autogrids with a cutout for FIB-milling.

#### Cryo-Focused ion beam (FIB) milling.

TEM-transparent lamellae were produced in a commercially available Aquilos2 dual-beam cryo-Focused Ion Beam and scanning electron microscope (cryo-FIB/SEM) instrument (Thermo Fisher Scientific). Intracellular lysosome fluorescence was observed using the integrated fluorescence light microscope (iFLM) of the Aquilos system. Fluorescent stacks were acquired using the 470 nm and 625 nm channels (4% laser intensity, 20ms exposure per slice, z-stack height of 6 μm, 15 slices). Correlation of the fiducial beads from the fluorescence 2D projection image with the grid’s respective SEM overview image was carried out in MAPS v.3.28 (Thermo Fisher Scientific).

Semi-automatic FIB-milling was done using AutoTEM v.2.4.2 (Thermo Fisher Scientific) at a milling angle of 8°, as described^69^. In short, the milling consisted of the following steps: i) rough milling at 1 nA to 1 μm thickness, (ii) 0.5 nA to 750 nm, and (iii) 0.3 nA to the 500 nm. Afterwards, the lamella was polished at a current of 50 pA to 150 nm) and 10 pA to 130 nm (with 0.2° overtilting). In some cases, remnants of the cell top surface with its organometallic layer had to be removed in addition to make the full tilt range in cryo-ET accessible.

#### Cryo-ET data acquisition and Processing.

TEM data acquisition was performed on a Krios G4 at 300 kV with Selectris X energy filter and Falcon 4i camera (Thermo Fisher Scientific) using SerialEM v.4.1 and v.4.2^70^. Tilt series were acquired at a nominal magnification of 64,000X (pixel size 1.971 Å) with a 10 eV energy slit using a dose-symmetric tilt scheme with an angular increment of 2°, a dose of ~2.4 e^−^/Å^2^ per tilt resulting in ~150 e/A^2^ for the 61 tilt of the complete tilt series, and a target defocus between −3 and −5 μm. Tilt series were collected ranging from −52° to +68° relative to the lamella pretilt. The positions for tilt series acquisition were determined by visual inspection of 8,700X magnification lamella montage maps. 10 frames per tilt were acquired and aligned in SerialEM during acquisition.

#### Tomogram reconstruction and segmentation.

The tilt series mrc stacks were corrected for dose exposure and projections of low-quality were removed manually. The tilt series was aligned using patch-tracking and reconstructed at bin4 using AreTomo (v.1.0.0)^71^ and IMOD (v.4.12.62)^72^. Tomogram denoising at bin4 (7.884Å/px) was done using cryoCARE^73^. All membranes in the tomograms were segmented with Membrain-Seg (https://github.com/teamtomo/membrain-seg)^74^ using the publicly available pretrained model (v10). For visualization purposes, all segmentations were manually touched up in Napari (doi:10.5281/zenodo.3555620) and rendered using ChimeraX v.1.10^75^.

### Software and Resources

The following software, packages, and resources were used for data analysis, visualization, and figure preparation:

#### R Environment:

R (v4.5.0; R Project for Statistical Computing); RStudio (v2025.05.0+496; Posit Software); bigstatsr (1.6.1); broom (1.0.8); Cairo (1.6.2); CalNetExploreR (0.1.0); car (3.1–2); circlize (0.4.16); ComplexHeatmap (2.25.2); ComplexUpset (1.3.3); cowplot (1.2.0); data.table (1.17.6); devtools (2.4.5); dplyr (1.1.4); factoextra (1.0.7); forcats (1.0.0); furrr (0.3.1); ggbiplot (0.6.2); ggdendro (0.2.0); ggdist (3.3.3); gghalves (0.1.4); gghighlight (0.5.0); ggplot2 (3.5.2); ggsci (2.9); ggVennDiagram (1.5.4); ggpmisc (0.6.1); ggpubr (0.6.1); ggraph (2.2.1); ggrepel (0.9.6); ggsignif (0.6.4); gptstudio (0.4.0); grid (4.5.0); gridExtra (2.3); igraph (2.1.4); irlba (2.3.5.1); limma (3.64.1); lintr (3.2.0); magick (2.8.7); msstats (4.10.0); NatParksPalettes (0.2.0); org.Hs.eg.db (3.21.0); patchwork (1.3.1); pheatmap (1.0.13); plotly (4.11.0); plyr (1.8.9); png (0.1.8); qs (0.27.3); readr (2.1.5); reshape2 (1.4.4); rlang (1.1.6); scales (1.4.0); signal (1.8.1); stringr (1.5.1); superheat (0.1.0); tidyplots (0.3.1); tidyverse (2.0.0); tidyr (1.3.1); timecourse (1.68.0); topGO (2.60.1); umap (0.2.10.0); UpSetR (1.4.0); uwot (0.2.3); viridis (0.6.5); zoo (1.8.14). Gene Ontology enrichment was performed using ShinyGO (Ge et al., 2020).

#### Python Environment:

Python (v3.12.2); cellposeSAM (v4.0.6); cellpose (≥4.0.5); torch (≥2.7.0); torchvision (≥0.22.0); pyqt6, qtpy, pyqt6-sip, pyqtgraph, superqt (latest versions); matplotlib, pandas, numpy, scikit-image, nd2, tifffile, tqdm (latest versions).

## Extended Data

## Supplementary Material

Supplement 1

Supplement 2 **Supplementary Table 3**: Generation of CRISPR-edited cell lines for interrogation of lysosomal storage disease gene function analysis. This file contains gRNA sequences as well as allele sequencing results for all edits examined. Additionally this file contains annotations of proteins to individual organelles

Supplement 3 **Supplementary Table 7**: TMT-based analysis of whole cell proteome of Control, *ASAH1*^−/−^ and *SMPD1*^−/−^ of iN, iDA at d23 of differentiation

**Supplementary Movie 1**: Tomogram of endolysosome in Control HeLa cells, corresponding to Fig. 1h **(left)**.

**Supplementary Movie 1**: Tomogram of endolysosome in *ASAH1*^−/−^ HeLa cells, corresponding to Fig. 1h **(right)**.

**Supplementary Table 1**: Label free lipidomics of WC and LysoIP of untagged, Control and *ASAH1*^−/−^ HeLa cells

**Supplementary Table 2:** TMT-based analysis of LysoIP sample proteomics of untagged, Control and *ASAH1*^−/−^ HeLa cells

**Supplementary Table 4**: nDIA whole cell proteomics of day 50 differentiated iN, iDA of LSD mutants

**Supplementary Table 5**: nDIA whole cell proteomics of day30 differentiated iN, iDA of select LSD mutants

**Supplementary Table 6**: TMT-based analysis of whole cell, soma and neuronal projection proteome of Control and *ASAH1*^−/−^ iN at d35 of differentiation

**Supplementary Table 8**: Combined nDIA whole cell proteomics of day 30, 50 and 70 differentiated iN, iDA of Control and *ASAH1*^−/−^

## Data Availability

Proteomic data (.RAW files) have been deposited into ProteomeXchange^76^. nDIA of iN/iDA day 30, 50 and 70: MSV000099237 (PXD068599). HeLa LysoIP TMT: PXD067219. iN iDA WC d23 TMT: PXD06720. iN axonal proteome d35 TMT: PXD067229. Lipidomics data is deposited on Metabolomics Workbench^77^: Study ID ST004217; http://dx.doi.org/10.21228/M8Z556. Raw Cryo-ET tomograms have been deposited at EMDB under accession numbers EMD-55210 (Control) and EMD-55211 (*ASAH1*^−/−^). A Key Resource Table containing reagents and materials, source data, segmentation models and supplementary datasets associated with this publication are available from Zenodo.org: 10.5281/zenodo.16733440.

## Code Availability

Scripts affiliated with this work is available on Github under the repository https://github.com/sauerkrausi/neuroLSD.
